# Bacterial Communities in Concrete Reflect Its Composite Nature and Change with Weathering

**DOI:** 10.1128/mSystems.01153-20

**Published:** 2021-05-04

**Authors:** E. Anders Kiledal, Jessica L. Keffer, Julia A. Maresca

**Affiliations:** a Department of Biological Sciences, University of Delaware, Newark, Delaware, USA; b Department of Civil and Environmental Engineering, University of Delaware, Newark, Delaware, USA; MIT

**Keywords:** concrete, alkali-silica reaction, low biomass, built environment, microbial communities, bioindicators

## Abstract

Concrete is an extreme but common environment and is home to microbial communities adapted to alkaline, saline, and oligotrophic conditions. Microbes inside the concrete that makes up buildings or roads have received little attention despite their ubiquity and capacity to interact with the concrete. Because concrete is a composite of materials which have their own microbial communities, we hypothesized that the microbial communities of concrete reflect those of the concrete components and that these communities change as the concrete ages. Here, we used a 16S amplicon study to show how microbial communities change over 2 years of outdoor weathering in two sets of concrete cylinders, one prone to the concrete-degrading alkali-silica reaction (ASR) and the other having the risk of the ASR mitigated. After identifying and removing taxa that were likely laboratory or reagent contaminants, we found that precursor materials, particularly the large aggregate (gravel), were the probable source of ∼50 to 60% of the bacteria observed in the first cylinders from each series. Overall, community diversity decreased over 2 years, with temporarily increased diversity in warmer summer months. We found that most of the concrete microbiome was composed of *Proteobacteria*, *Firmicutes*, and *Actinobacteria*, although community composition changed seasonally and over multiyear time scales and was likely influenced by environmental deposition. Although the community composition between the two series was not significantly different overall, several taxa, including *Arcobacter*, *Modestobacter*, *Salinicoccus*, *Rheinheimera*, *Lawsonella*, and *Bryobacter*, appear to be associated with ASR.

**IMPORTANCE** Concrete is the most-used building material in the world and a biologically extreme environment, with a microbiome composed of bacteria that likely come from concrete precursor materials, aerosols, and environmental deposition. These microbes, though seeded from a variety of materials, are all subject to desiccation, heating, starvation, high salinity, and very high pH. Microbes that survive and even thrive under these conditions can potentially either degrade concrete or contribute to its repair. Thus, understanding which microbes survive in concrete, under what conditions, and for how long has potential implications for biorepair of concrete. Further, methodological pipelines for analyzing concrete microbial communities can be applied to concrete from a variety of structures or with different types of damage to identify bioindicator species that can be used for structural health monitoring and service life prediction.

## INTRODUCTION

Concrete is a uniquely harsh environment characterized by high alkalinity and salinity and low water activity. In outdoor structures, it is also subject to large fluctuations in temperature and moisture. Due to its strength, resistance to weathering, and low cost, concrete is the most widely used building material in the world (1). It is, therefore, a very common environment, and despite the tough conditions, bacteria are known to live in and on concrete (2–11).

When concrete is poured, its pH is ∼12.5, higher than most known naturally alkaline environments (12, 13) and comparable to that of leachate from steel slag or bauxite and Solvay wastes (14). Highly alkaline environments like concrete present metabolic and physical challenges to the microbes inhabiting them, including a reversed proton gradient, with implications for ATP production, enzyme inactivation, and instability of membranes and DNA, among others (15). Microbe-concrete relationships have been extensively studied in the specific contexts of concrete degradation (3–9, 16–18) and biorepair (19). In these cases, the microbes that alter the concrete structure are introduced from outside after the structure has been poured. However, very little is known about the microbes that inhabit ordinary concrete. We hypothesized that the concrete microbiome comes from the precursor materials and that it is similar to that of other high-pH environments, like alkaline soils and soda lakes.

We further predicted that concrete mixes with different chemical properties would have different microbial communities, so we compared the microbial communities of a concrete mix prone to alkali-silica reaction (ASR) and one for which the risk of ASR had been mitigated with fly ash. ASR is a concrete-degrading chemical reaction of global concern that occurs between alkali hydroxides from cement powder and silica in the fine and large aggregates that constitute most of concrete’s mass (20). ASR results in a silicate gel that expands when hydrated, creating internal pressure and extensive map cracking that significantly shortens the life span of affected structures. To prevent ASR, the Delaware Department of Transportation adds fly ash to the concrete mix, a common practice (21). In addition to reducing the probability of ASR, fly ash reacts with lime, and the reaction products fill concrete pores, particularly larger ones most prone to water infiltration, decreasing concrete porosity (22, 23). Because ASR alters both the chemistry of the concrete and its structure, allowing more infiltration by water and waterborne microbes and chemicals (20, 24), while fly ash reduces porosity and excess alkalinity (21, 25), we expected the microbial communities in these two types of concrete to diverge from each other over time.

We expected analysis of these communities to be complicated, because concrete is a low-biomass environment (2, 26), and such environments are particularly susceptible to laboratory contamination. Contaminant DNA represents a higher proportion of total DNA in low-biomass systems than it does in a higher-biomass environment and thus has more power to obscure noncontaminant sequences or influence interpretation of the data (27). For this reason, it is important to use rigorous methods to identify and categorize as many potential contaminants and their likely sources as possible. An aggressive approach to identifying and removing contaminants increases confidence that the real concrete microbiome, not reagent or laboratory microbiomes, can be described.

Our previous work showed that cultivable bacteria are present in concrete and that microbial DNA can be extracted from concrete and analyzed, providing a snapshot of the bacteria living in and on concrete a year after pouring (2). Here, we investigated how precursor materials, probability of ASR, time, weather, and laboratory contamination influence concrete microbial communities. We addressed these questions using 16S amplicon sequencing to characterize, over 2 years, the microbial communities of two series of concrete cylinders made with different mix designs to confer or prevent ASR and the precursor materials used in their preparation.

## RESULTS

### Concrete cylinder preparation and sampling.

**FIG 1:**
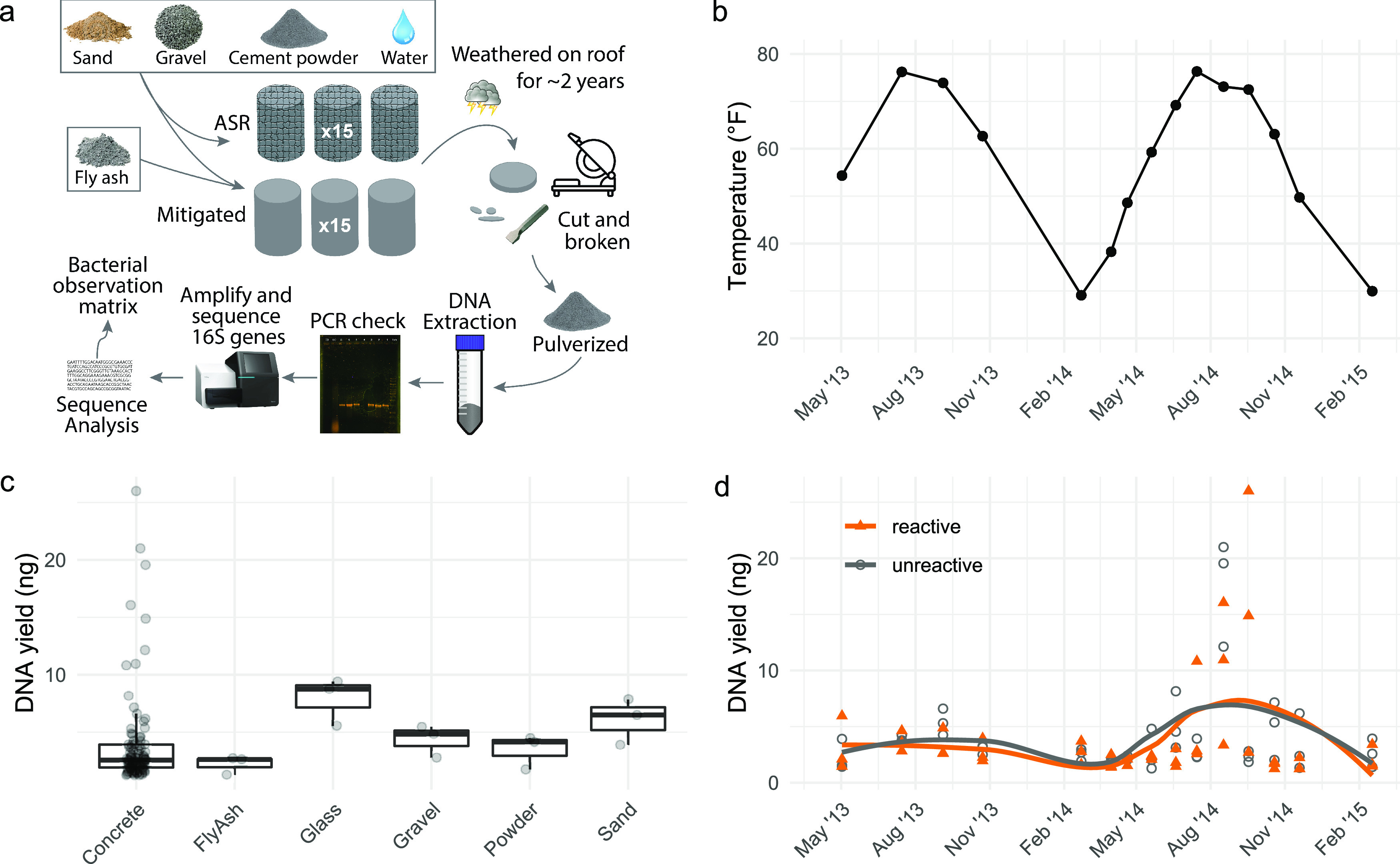
Experimental design, sample collection, and DNA extraction. (a) Two series of concrete cylinders, one prone to the ASR and the other having the risk of the ASR mitigated with fly ash, were placed on a campus roof for 2 years. Every 4 to 8 weeks, one cylinder of each type was archived. DNA was extracted from triplicate samples of pulverized concrete and precursor materials and sent for 16S amplicon sequencing with primers 357F and 806R (29, 30). (b) Temperatures throughout the sampling period. Each point represents a sample collection date, and reported temperatures are the mean temperature of the 30 days preceding collection. (c) DNA extraction yields from 5 g of material obtained using the protocol from reference 2. Triple-sterilized glass beads served as a negative control. Quantification was performed with a Qubit double-stranded-DNA high-sensitivity fluorometric assay. Differences in extraction efficiency may contribute to apparent differences between materials. (d) DNA yield from 5 g of concrete for each concrete sample in the series. DNA yields were higher for samples collected during the second summer.

To determine how microbial communities change as concrete weathers, and to test the effects of ASR reactivity on community composition, we prepared two series of concrete cylinders which were weathered outdoors on a rooftop for ∼2 years. One cylinder from each series was collected approximately every 6 weeks and sampled by slicing an internal section from each (Fig. 1a). Although 2 years is short on the time scale of ASR development, this time frame allows the microbial communities to be exposed to multiple annual temperature and precipitation cycles (Fig. 1b) and is long enough to see the early effects of ASR damage (28). DNA was extracted from the concrete samples, the precursor materials used in the concrete mixes, and triple sterilized glass beads, as the negative control (Fig. 1c). DNA yields ranged from 1.25 ng to 26 ng of DNA from 5 g of starting material, with the highest yields obtained during the second summer (Fig. 1d).

### 16S amplification, sequencing, and quality filtering.

Using primers 357F and 806R (29, 30), the V3-V4 region of the 16S gene was amplified from 105 samples, representing 90 concrete samples (15 time points × 2 ASR conditions × 3 replicates) and triplicate samples of each constituent material and negative-control glass beads (see table S1 at doi.org/10.6084/m9.figshare.14211038). A total of 9.4 million 300-bp paired-end Illumina MiSeq amplicon reads were obtained (mean, 89,743 reads per sample). Following initial primer trimming and quality filtering with Cutadapt, 8.6 million high-quality reads were retained. DADA2 denoising, chimera filtering, and read joining performed with the QIIME 2 platform (31, 32) resulted in 5.1 million observations (mean, 48,600 per sample) of 6,924 unique amplicon sequence variants (ASVs) (see table S1 at doi.org/10.6084/m9.figshare.14211038). ASVs that were identical in their overlapping region but differed in length were merged with vsearch clustering at 100% similarity, resulting in 6,898 unique ASVs (33). Finally, 31 ASVs of unexpectedly short length (<400 bp) were discarded, resulting in 48,526 mean reads per sample of 6,867 unique ASVs. For phylogenetic analyses, ASV representative sequences were inserted into the SILVA reference phylogeny (release 128) (34–36). All sequences that passed the >400-bp length filter were successfully inserted into the reference tree.

### Contaminant identification and removal.

Contaminant ASVs are present in nearly all 16S samples (27, 37) and can dominate results in low-biomass environments (38), so several established and custom techniques were used to classify the observed taxa as concrete-associated or contaminant. The *prevalence* method of the *decontam* R package (39) was used to identify 181 ASVs statistically more likely to be found in negative controls (glass beads) than samples (see table S2 at doi.org/10.6084/m9.figshare.14211038; also, see Fig. S1a in the supplemental material). The *frequency* method of *decontam*, which identifies ASVs whose relative abundance is inversely correlated with sample DNA concentration, was not applied because it is less reliable for very-low-biomass samples (39) and because our DNA concentrations were correlated with other sample metadata, such as season/temperature.

10.1128/mSystems.01153-20.4 FIG S1 Euler plot of contaminants identified by each method. (A) This Euler plot summarizes the number of contaminants determined by each method and combination of methods. The majority of contaminants were identified as such by multiple methods. SPARCC, SPIEC-EASI, and propr represent the correlation-based methods, which are compared with the *decontam* R package’s *prevalence* method. ASVs observed in negative controls (dashed border) are included for comparison only, as they were not used alone as a decontamination criterion, although nearly all were determined to be contaminants. The correlation-based methods had significant agreement, identifying significantly more ASVs as contaminants than *decontam*. However, 163 contaminants were uniquely determined by *decontam*’s prevalence method. (B) This Euler plot summarizes the number of each type of contaminant and the overlap of contaminant types. ASVs observed in negative controls (blue circle with dashed border) are included for comparison, although this alone was not a qualification for contaminant classification. The *decontam* R package’s prevalence method is based on ASV observation in negative controls, which explains their significant overlap. Negative controls generally catch reagent contamination, which likely explains their overlap with reagent contaminants identified by the correlation-based methods. Although mostly a distinct group, there is a small amount of overlap between reagent contaminants and the lab and secondary contaminants (contaminants correlated with both lab and reagent contaminants) identified by the correlation methods. Download FIG S1, PDF file, 0.2 MB. Copyright © 2021 Kiledal et al. 2021 Kiledal et al. https://creativecommons.org/licenses/by/4.0/ This content is distributed under the terms of the Creative Commons Attribution 4.0 International license.

Reagent contaminants were identified with a correlation approach. ASV-ASV interaction networks were determined with SPARCC (40) implemented in FastSpar (41), SPIEC-EASI (42), and the *propr* R package (43). In this approach, core groups of highly intercorrelated contaminants were first identified for two types of contamination: reagent and laboratory environment. The core group of reagent contaminants was identified as highly intercorrelated ASVs present in negative-control samples with more strong positive than strong negative correlations with other negative-control ASVs (scripts available at github.com/MarescaLab/concrete_series). Reagent contaminants determined with SPARCC were also required to have a mean correlation greater than 0.3 with other negative-control ASVs. All other ASVs were then screened for correlations with this list of reagent contaminants and classified as contaminants if they exceeded cutoffs for the net number of positive correlations and/or mean correlation with the core reagent contaminants (see “Pairwise ASV comparisons” and “Contaminant identification”). Six hundred fifty-four ASVs were identified as suspected reagent contaminants with this method, of which 359 were uniquely identified by this method (see table S2 at doi.org/10.6084/m9.figshare.14211038; Fig. S1b). The 149 ASVs observed only in negative controls were also classified as reagent contaminants. The most abundant reagent contaminants belong to the *Burkholderiaceae* (*Betaproteobacteria*), while ASVs from *Enterococcus*, *Methylobacterium*, *Sphingomonas*, and *Bradyrhizobium* were also abundant reagent contaminants (Fig. S2 and S3).

10.1128/mSystems.01153-20.5 FIG S2 Contaminant taxa composition and relative abundance. The relative abundance (node size) of contaminant taxa pooled across all concrete samples, with the central bacterial node equal to a size of one representing all contaminants. Contaminants were determined with several methods, including the *prevalence* methods of the *decontam* R package, BLAST similarity to known lab contaminants, and correlation-based methods. Higher phylogenetic ranks are more central in the tree, and lower ranks (species) are found at the periphery. The most abundant contaminants belong to *Burkholderiaceae*, *Enterobacteriaceae*, *Enterococcaceae*, *Pseudomonadales*, and *Rhizobiales*. Download FIG S2, PDF file, 0.3 MB. Copyright © 2021 Kiledal et al. 2021 Kiledal et al. https://creativecommons.org/licenses/by/4.0/ This content is distributed under the terms of the Creative Commons Attribution 4.0 International license.

10.1128/mSystems.01153-20.6 FIG S3 Contaminant taxa relative abundance by type. This heat tree matrix compares the taxonomic composition and relative abundance of different contaminant types. The large grey tree in the bottom left is provided for taxonomic reference and is repeated for each pairwise tree without labels. The color of nodes and edges represents the log_2_ ratio between each pair. Download FIG S3, PDF file, 0.2 MB. Copyright © 2021 Kiledal et al. 2021 Kiledal et al. https://creativecommons.org/licenses/by/4.0/ This content is distributed under the terms of the Creative Commons Attribution 4.0 International license.

Contaminants introduced from nonreagent laboratory sources like air, surfaces, or human handling were identified in a similar manner. Several strains researched in our laboratory, such as Rhodoluna lacicola, were unexpectedly observed in the data and are likely contaminants. Nineteen ASVs with >99% similarity to 5 likely lab contaminants (as determined via BLAST alignment [44]) were identified as contaminants (see table S2 at doi.org/10.6084/m9.figshare.14211038). We identified 167 additional contaminants by correlation with these 19 ASVs (scripts available at github.com/MarescaLab/concrete_series). Of these 167 contaminants, 147 were uniquely identified by strong correlation with known contaminants while 20 were also identified by other methods. The most abundant lab contaminants were enterobacteria in the genera *Escherichia-Shigella* and *Pantoea*. Other putative abundant lab contaminants included ASVs from *Microbacteriaceae*, *Pseudomonas*, *Sediminibacterium*, *Pedobacter*, *Exiguobacterium*, *Planococcus*, *and Bacillus*.

Finally, remaining ASVs were screened against a master list of reagent and laboratory contaminants identified up to this point, identifying 98 additional contaminants using correlation metric-specific cutoffs. (see table S2 at doi.org/10.6084/m9.figshare.14211038; Fig. S1b). Many of these putative contaminants belong to taxa identified as common contaminants in other studies (45), such as *Sphingomonas*, *Burkholderiaceae*, *Enterobacteriaceae*, *Acidibacter*, *Planococcaceae*, *Bacillus*, and *Micrococcaceae* (Fig. S3). Certain species of *Bacteroidia*, *Pedobacter*, and *Vibrionimonas* were also identified as contaminants this way.

In total, 1,112 of 6,867 ASVs were determined to be contaminants. The most abundant contaminants were *Proteobacteria* (*Beta-*, *Gamma-*, and *Alphaproteobacteria*, in decreasing order of abundance) and *Firmicutes* (Fig. S2; see table S3 at doi.org/10.6084/m9.figshare.14211038). Removal of potential contaminant reads left 547,573 observations of 5,755 ASVs, an average of 5,368 observations per sample. Only ∼15% of reads were retained because of our aggressive contaminant identification approach; however, there was little impact on the observed diversity because in about 85% of samples, >50% of the removed reads were from only a few (<5) high-abundance contaminants.

Prior to decontamination, we observed low replicate similarity (1 − generalized [0.5] UniFrac distance). The mean similarity within concrete replicates was 0.480, not significantly higher than the mean between-sample similarity of 0.466 (one-sided Welch’s *t* test; *P* = 0.166). This was also true following decontamination where the mean within-replicate similarity was 0.340 and the between-replicate similarity was 0.332 (one-sided Welch’s *t* test; *P* = 0.111). Decontamination decreased within-replicate similarity (one-sided Welch’s *t* test; *P* = 2.634 × 10^−15^).

### Concrete microbial community composition.

**FIG 2:**
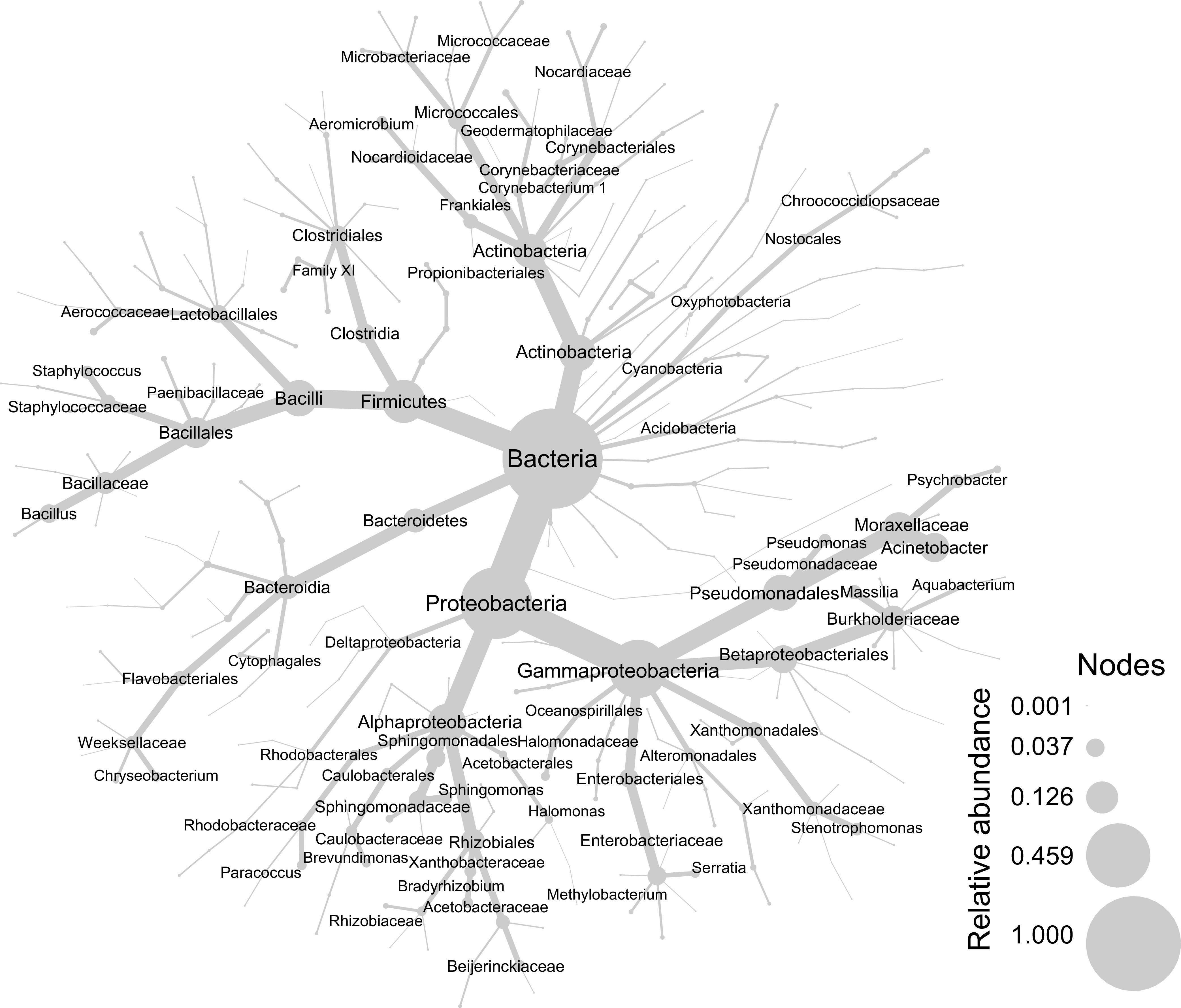
Concrete bacterial community relative abundance. The relative abundances of bacterial taxa observed in all concrete samples from the time series are indicated by node size and edge thickness. Higher phylogenetic ranks are more central in the tree, and lower ranks, such as species, are found at the periphery. *Proteobacteria*, *Firmicutes*, and *Actinobacteria* are the most abundant phyla observed. ASVs determined to be contaminants have been removed.

To broadly characterize the microbial communities in concrete, all concrete cylinder samples were pooled after removal of contaminants. Overall, 50% of sequences were classified at the phylum level as *Proteobacteria*, 19% as *Firmicutes*, 14% as *Actinobacteria*, 7% as *Cyanobacteria*, 5% as *Bacteroidetes*, ∼1% as *Acidobacteria*, and ∼0.5% as *Planctomycetes*, with other phyla each representing <0.5% of observations (Fig. 2). No *Archaea* were observed in this data set. All percentages referenced in this section are of the total ASV observation count (547,573) after decontamination for all taxa.

Of the *Proteobacteria*, *Gammaproteobacteria* were the most abundant (∼34% of all reads), in part due to the pseudomonad genera Acinetobacter (9%, the most abundant genus observed), *Pseudomonas* (∼2%), and *Psychrobacter* (∼1.7%). Other groups of *Gammaproteobacteria* were also abundant, particularly the families *Enterobacteriaceae* (∼5%), *Xanthomonadaceae* (∼2%), and *Halomonadaceae* (∼1%). Nearly 9% of sequences were classified as *Betaproteobacteriales*, of which most belong to the family *Burkholderiaceae* (∼8%). *Alphaproteobacteria* (∼15%) were also observed, particularly those belonging to the orders *Rhizobiales* (∼5%) and *Sphingomonadales* (∼4%).

Gram-positive taxa account for ∼33% of all reads. Almost 19% of sequences were classified as *Firmicutes*, which can form spores, potentially allowing them to survive in dormant states in the harsh concrete environment. Most of these belong to the order *Bacillales* (∼10%), such as members of the genera *Bacillus* (∼4%) and *Staphylococcus* (∼2%). Nearly 5% of sequences also belonged to the order *Lactobacillales*. Actinobacteria account for ∼14% of sequences, spread across several families: *Nocardiaceae* (2.0%), *Dietziaceae* (2.0%), *Corynebacteriaceae* (1.4%), *Microbacteriaceae* (1.4%), *Micrococcaceae* (1.2%), *Nocardiaceae* (0.99%), and *Geodermatophilaceae* (0.83%).

Three additional phyla each represented more than 1% of all sequences: *Cyanobacteria* (∼7%), *Bacteroidetes* (∼5%), and *Acidobacteria* (∼1%). Many of the cyanobacteria were classified as chloroplasts (∼6%) and were likely introduced from pollen or other plant material.

### Precursor community composition.

**FIG 3:**
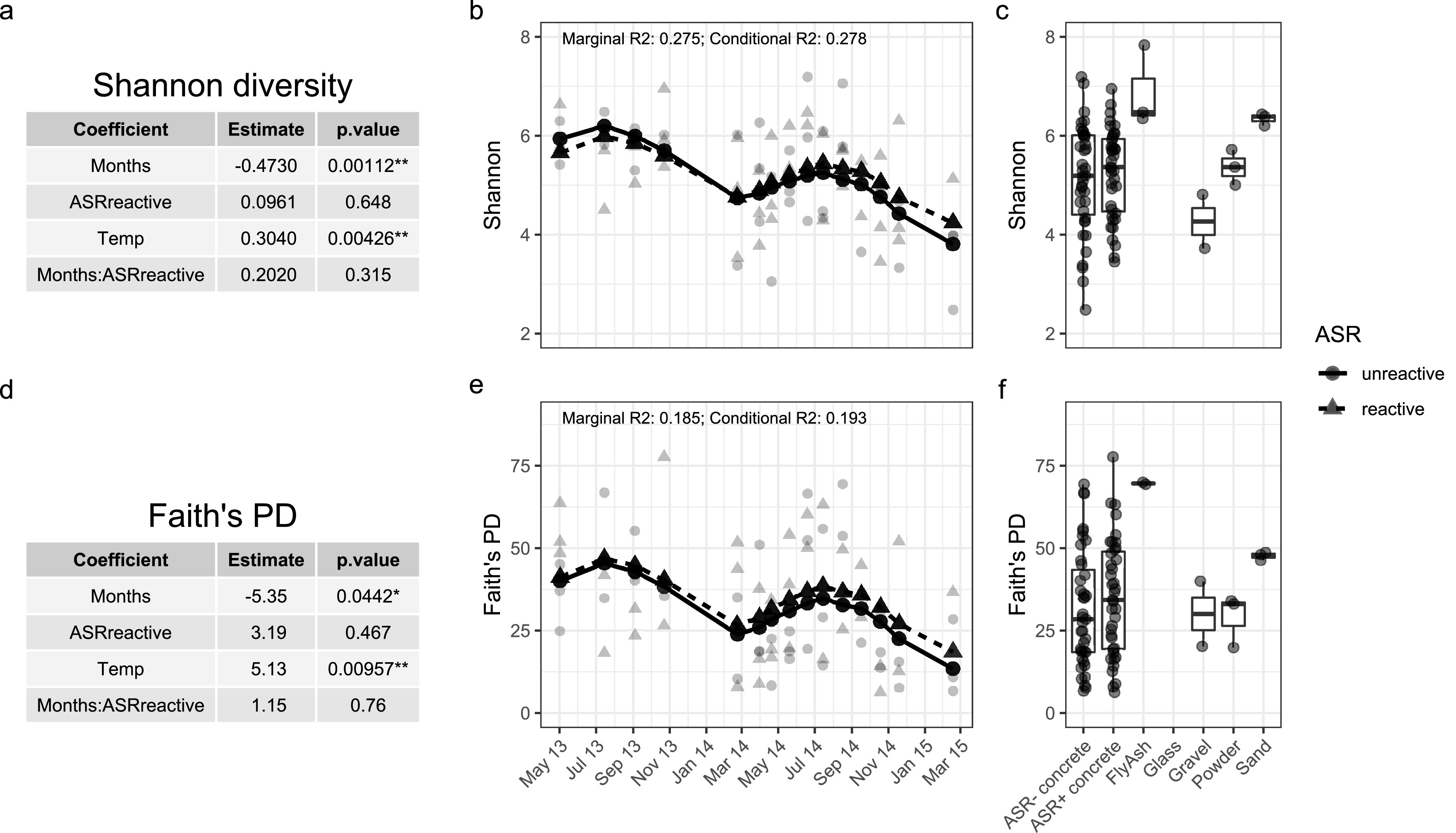
Within-sample diversity over time. Two metrics of bacterial diversity, Shannon diversity (a to c) and Faith’s phylogenetic diversity (d to f), show similar trends in both decontaminated cylinder series. In panels b and e, light points are the observed sample values, while connected dark points were predicted with a linear mixed-effect model. (a and d) Coefficients for both models show that time (months) has a significant negative effect on diversity, while temperature has a significant positive effect (*, *P* ≤ 0.05; **, *P* ≤ 0.01). ASR reactivity appears to have little effect on bacterial diversity, although reactive concrete is associated with (nonsignificant) increased diversity for both metrics. The interaction effect of time and ASR reactivity indicates that over time, more diversity is associated with ASR reactivity; however, this cannot be considered significant, since the effect of ASR reactivity alone was nonsignificant. (c and f) Concrete sample diversity compared to precursor material diversity.

Prior to decontamination, higher diversity (Faith’s phylogenetic diversity [PD]) was observed in sand and fly ash than concrete, while the diversity of negative controls (glass), cement powder, and gravel was similar to the median diversity of concrete samples (Fig. S4). Diversity patterns after decontamination were similar, with generally greater bacterial diversity observed in fly ash and sand samples than in concrete and diversity similar to that in concrete observed in gravel and powder samples (Fig. 3). Shannon and Faith’s diversity metrics generally showed similar differences between groups, although decontaminated gravel samples and cement powder samples before decontamination had comparatively low Shannon values.

10.1128/mSystems.01153-20.7 FIG S4 Raw within-sample diversity. Two metrics of bacterial diversity, Shannon diversity (a to c) and Faith’s phylogenetic diversity (d to f) show trends in both undecontaminated cylinder series. In panels b and e, light points are observed sample values while connected dark points were predicted with a linear mixed-effect model. Concrete sample diversity is compared to precursor material diversity in panels c and f. Download FIG S4, PDF file, 0.1 MB. Copyright © 2021 Kiledal et al. 2021 Kiledal et al. https://creativecommons.org/licenses/by/4.0/ This content is distributed under the terms of the Creative Commons Attribution 4.0 International license.

Each of the precursor materials had indicator taxa that were exclusive to that material. Indicator values (IndVal), a measure of exclusiveness, were calculated for these taxa as the product of relative observation frequency (specificity) and likelihood of observation (sensitivity) with a correction for unequal group sizes (46). Gravel communities, the least diverse of the precursors, were composed primarily of Acinetobacter, *Pelotomaculum*, *Bacillus*, *Aeromicrobium*, and *Burkholderia* (see table S4 at doi.org/10.6084/m9.figshare.14211038). *Nosocomiicoccus* was an indicator for gravel samples (IndVal = 0.82, *P* = 0.02), although its relative abundance—the proportion of all observations—was low (6 × 10^−5^). Cement powder bacterial communities consisted primarily of *Pantoea*, *Burkholderia*, Acinetobacter, *Psychrobacter*, *Streptococcus*, *Staphylococcus*, *Chryseobacterium*, and *Corynebacterium* (see table S4 at doi.org/10.6084/m9.figshare.14211038). *Nocardia* was an indicator of cement powder samples (IndVal = 0.996, *P* = 0.001, rel.abund ∼ 0.0003). Fly ash microbial communities consisted primarily of *Paracoccus*, *Hydrogenophaga*, *Bacillus*, *Thiobacillus*, and *Nocardioides* (see table S4 at doi.org/10.6084/m9.figshare.14211038). Several genera were indicators of fly ash, including *Thermithiobacillus* (IndVal = 1, *P* = 0.001, rel.abund ∼ 0.005), *Meiothermus* (IndVal = 0.998, *P* = 0.001, rel.abund ∼ 0.02), *Truepera* (IndVal = 0.988, *P* = 0.005, rel.abund ∼ 0.02), and uncultured members of “*Candidatus* Kaiserbacteria” (IndVal = 1, *P* = 0.001, rel.abund ∼ 0.01).

Of the precursors, sand microbial communities were the most different from those of concrete. We observed more *Alphaproteobacteria* in sand samples (see table S4 at doi.org/10.6084/m9.figshare.14211038), specifically *Sphingomonadaceae* like *Rubritepida* (rel.abund ∼0.05), *Porphyrobacter* (rel.abund ∼0.03), *Sphingoaurantiacus* (rel.abund ∼0.002), and *Sandaracinobacter* (rel.abund ∼0.0004) (all indicator values > 0.99, *P* < 0.01). Other taxa with high indicator values for sand were *Bacteroidia*, including *Rhabdobacter* (IndVal = 0.97, *P* = 0.02, rel.abund ∼0.06) and *Cnuella* (IndVal = 0.99, *P* = 0.001, rel.abund ∼0.05), and the *Gammaproteobacteria Alishewanella* (IndVal = 0.99, *P* = 0.001, rel.abund ∼0.07) and *Lysobacter* (IndVal = 0.98, *P* = 0.007, rel.abund ∼0.02). *Gemmatirosa* (IndVal = 0.99, *P* = 0.001, rel.abund ∼0.02) and several *Verrucomicrobia*, such as *Prosthecobacter* (IndVal = 1, *P* = 0.001, rel.abund ∼0.004), also had high indicator values for sand.

### Influence of precursor materials on concrete microbial communities.

**FIG 4:**
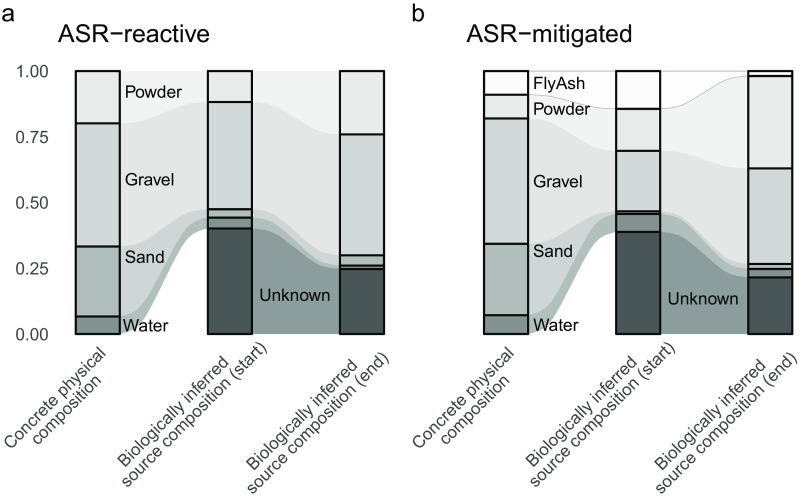
Influence of precursors on concrete bacterial communities. Concrete composition by weight (a and b, left) compared to the mixing proportion of source materials inferred by comparing concrete microbial communities to precursor material communities (a and b, right). Using sequenced microbial community information for concrete samples (“sinks”) and precursor materials (“sources”), the SourceTracker2 tool was used to infer the proportion of bacteria in concrete sinks originating from each precursor source. SourceTracker also predicts a proportion of bacteria from an unknown source. Inferred proportions are for the first concrete samples in each series, archived prior to weathering. Unlike the other precursor materials, sequence data were not directly obtained from the water used for concrete production (Newark, DE, tap water). Instead, tap water samples from multiple utilities in the eastern United States were used (48).

SourceTracker analysis (47) was used to assess the influence of the concrete constituent material microbial communities on bacteria in the first (*t*_0_; May 2013) and last (*t*_end_; February 2015) concrete samples (Fig. 4). Using microbial community data from concrete precursor materials (sources), SourceTracker identified the probable fraction of microbes in the concrete cylinders coming from each precursor material and also estimated the contribution of an “unknown” source (mean contribution to reactive and unreactive concrete: *t*_0_ ∼ 39%, *t*_end_ ∼ 23%). Precursor sources considered in this analysis were fly ash (*t*_0_ ∼ 7%, *t*_end_ ∼ 1%), the large aggregate gravel (*t*_0_ ∼ 32%, *t*_end_ ∼ 41%), the fine aggregate sand (*t*_0_ ∼ 2%, *t*_end_ ∼ 3%), and cement powder (*t*_0_ ∼ 14%, *t*_end_ ∼ 30%). Amplicon sequence data were not obtained for the water used in cylinder preparation (Newark, DE, tap water) and was instead approximated with data from multiple water utilities in the eastern United States published in reference 48. Leave-one-out cross validation was performed (Fig. S5). This SourceTracker analysis suggests that precursor materials contribute a considerable (>50%) portion of the concrete microbiome in early samples, which represents an even larger portion of the bacterial community in later samples (Fig. 4). Gravel had the greatest contribution. Interestingly, sand was found to have little influence on the composition of the concrete microbial community, though it is a major constituent material of concrete and had high bacterial diversity (Fig. 3). In the ASR-mitigated concrete, gravel had a smaller contribution while fly ash and cement powder had larger predicted contributions to the microbial community (Fig. 4). Over time, the proportion of microbes predicted to be of unknown origin decreased, while similarity to gravel and cement powder communities increased.

10.1128/mSystems.01153-20.8 FIG S5 Precursor SourceTracking leave-one-out cross validation. Leave-one-out validation of SourceTracking analysis shows the potential for misclassification between sources. Higher values indicate that the sink on the *y* axis is more similar to the source on the *x* axis. The microbial communities of gravel, cement powder, and sand have the most similarity to those of fly ash, while microbial communities from fly ash and tap water have the most in common with sand communities. Download FIG S5, PDF file, 0.1 MB. Copyright © 2021 Kiledal et al. 2021 Kiledal et al. https://creativecommons.org/licenses/by/4.0/ This content is distributed under the terms of the Creative Commons Attribution 4.0 International license.

### Time and season affect diversity and community composition.

We analyzed the bacterial diversity in concrete and precursor materials and how diversity in concrete changed over time. Within-sample (alpha) diversity metrics, Shannon and Faith’s phylogenetic diversity, were calculated with QIIME 2 on decontaminated individual concrete samples subsampled to 1,000 observations (Fig. 3). Shannon diversity summarizes both richness and evenness; however, because it can obscure absolute richness, Faith’s phylogenetic diversity was also included. Linear mixed-effect models were applied to both metrics, yielding similar results: diversity decreased over time (months of weathering) (Shannon’s estimate = −0.47, *P* = 0.001; Faith’s PD estimate = −5.35, *P* = 0.04), with seasonal increases associated with warmer temperatures (Shannon’s estimate = 0.3, *P* = 0.004; Faith’s PD estimate = 5.13, *P* = 0.01). Probability of ASR did not have a significant effect on observed diversity, although reactive samples had slightly higher diversity than mitigated samples with both metrics (Shannon’s estimate = 0.096, *P* = 0.6; Faith’s PD estimate = 3.2, *P* = 0.5) (Fig. 3). To understand the effects of decontamination, alpha diversity was also calculated from raw (before decontamination) data (Fig. S4). Removing laboratory contaminants strengthened the apparent effect of season on diversity, though the effect was present in the raw data.

Permutational multivariate analysis of variance (PERMANOVA) models (49, 50) were used to assess environmental influences on community composition. Like alpha diversity, time (months of weathering) and temperature were associated with significant changes (*P* < 0.05) in overall community structure as determined with PERMANOVA performed on generalized (0.5) UniFrac distances (Table 1). This significant effect was observed when added sequentially to the model (distance ∼ temperature + time + ASR) and also when the marginal effect of each term was considered. The marginal effect of time was greater than that of temperature, indicating that the time since pouring has a greater impact than temperature. No significant difference in overall community composition was observed between ASR-reactive and ASR-unreactive cylinders. Dispersion was also assessed, since it can confound PERMANOVA models, but no significant differences were found (Table 1). Other distance metrics yielded consistent results (Table S1), as did Mantel matrix correlation tests, although the Mantel tests did not find time alone to be significant (Table S2). PERMANOVA on predecontamination data also revealed significant time and temperature effects (Table S3).

**TABLE 1:** PERMANOVA results for the generalized UniFrac metric^*a*^

Term testing	Coefficient	*R*^2^	*F*	*P* value	Dispersion *P* value
Sequential	Temp	0.024	1.851	0.003**	0.891
	Mo	0.025	1.972	0.002**	0.891
	ASR	0.01	0.802	0.896	0.671
Marginal	Temp	0.02	1.607	0.015*	0.891
	Mo	0.025	1.939	0.001***	0.891
	ASR	0.01	0.802	0.898	0.671

a PERMANOVA models (distance ∼ temperature + months + ASR) were computed with the *adonis2* function of the *vegan* R package for generalized (0.5) UniFrac distances. The effect of terms was tested both sequentially and marginally. For both models, temperature and time had significant (*, *P* ≤ 0.05; **, *P* ≤ 0.01; ***, *P* ≤ 0.005) associations with observed bacterial community differences. Pseudo-*F* ratios compare the total sum of squared dissimilarities between groups to those within groups, a measure of group separation, with larger *F* ratios indicating greater differences between groups. Dispersion was also assessed with the *betadisper* function of the *vegan* R package for each distance/term pair because of potential for confounding. No significant differences in dispersion were observed.

10.1128/mSystems.01153-20.1 TABLE S1 PERMANOVA community composition tests with Bray-Curtis distances. PERMANOVA models (distance ∼ temperature + months + ASR) were computed with the *adonis2* function of the *vegan* R package for Bray-Curtis distances. The effect of terms was tested both sequentially and marginally. For both models, temperature and time had significant (*, *P* ≤ 0.05; **, *P* ≤ 0.01; ***, *P* ≤ 0.005) associations with observed bacterial community differences. Pseudo-*F* ratios compare the total sum of squared dissimilarities between groups to those within groups, a measure of group separation, with larger *F* ratios indicating greater differences between groups. Dispersion was also assessed with the *betadisper* function of the *vegan* R package for each distance/term pair because of potential for confounding. No significant differences in dispersion were observed. Download Table S1, PDF file, 0.09 MB. Copyright © 2021 Kiledal et al. 2021 Kiledal et al. https://creativecommons.org/licenses/by/4.0/ This content is distributed under the terms of the Creative Commons Attribution 4.0 International license.

10.1128/mSystems.01153-20.2 TABLE S2 Correlation of time and temperature with community level changes. Mantel matrix correlation tests were performed for time, temperature, and both time and temperature with generalized (0.5) UniFrac and Bray-Curtis sample distance/dissimilarity matrices. Mantel tests with the Pearson metric were performed in the *vegan* R package, and significance was determined with 999 permutations. With both distance metrics, temperature and combined time and temperature were significant (*, *P* ≤ 0.05; **, *P* ≤ 0.01; ***, *P* ≤ 0.005), while time alone (months) was not. Download Table S2, PDF file, 0.1 MB. Copyright © 2021 Kiledal et al. 2021 Kiledal et al. https://creativecommons.org/licenses/by/4.0/ This content is distributed under the terms of the Creative Commons Attribution 4.0 International license.

10.1128/mSystems.01153-20.3 TABLE S3 PERMANOVA tests for concrete samples before decontamination. PERMANOVA models (distance ∼ temperature + months + ASR) were computed with the *adonis2* function of the *vegan* R package for generalized (0.5) UniFrac sample distances calculated before decontamination. The effect of terms was tested both sequentially and marginally. For both models, temperature and time had significant (*, *P* ≤ 0.05; **, *P* ≤ 0.01; ***, *P* ≤ 0.005) associations with observed bacterial community differences. Pseudo-*F* ratios compare the total sum of squared dissimilarities between groups to those within groups, a measure of group separation, with larger *F* ratios indicating greater differences between groups. Dispersion was also assessed with the *betadisper* function of the *vegan* R package for each distance/term pair because of potential for confounding. No significant differences in dispersion were observed. Download Table S3, PDF file, 0.1 MB. Copyright © 2021 Kiledal et al. 2021 Kiledal et al. https://creativecommons.org/licenses/by/4.0/ This content is distributed under the terms of the Creative Commons Attribution 4.0 International license.

### Taxonomic changes over time.

Large-scale changes in diversity and community composition are often driven by changes in the presence and/or abundance of particular taxa. Therefore, each pattern observed with alpha- and beta-diversity metrics should have groups of bacteria following the same pattern. The changes in diversity associated with time and temperature reflect changes in patterns of abundance of both common and rare taxa.

**FIG 5:**
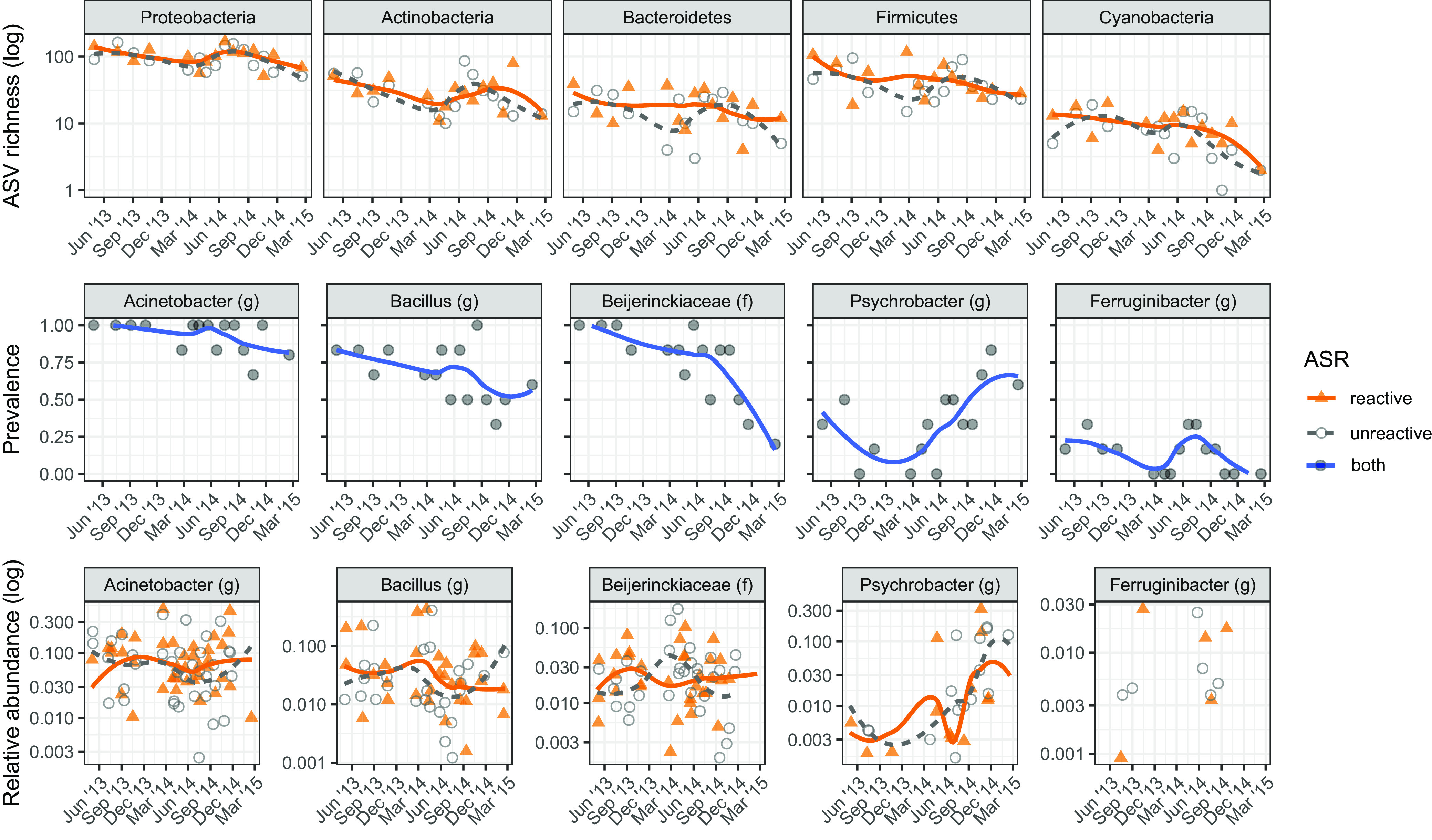
Taxon changes over time. Changes in the diversity (top), prevalence (middle), and relative abundance (bottom) of certain taxa observed over the 2 years during which concrete cylinders were weathered. The diversity (number of ASVs) of the five most abundant phyla is shown in the top row; they generally exhibit the same trends of decreasing diversity with seasonally increased diversity in summer seen for overall diversity (Fig. 3). Several examples of prevalence (the proportion of samples in which a group appears) at each time point are shown, including for the two most abundant genera, Acinetobacter and *Bacillus*, whose prevalence roughly mirrors prevailing diversity patterns. Logistic regression of presence/absence across the series was used to identify taxa with increasing (*Psychrobacter*) or decreasing (*Beijerinckiaceae*) prevalence. Seasonally associated taxa were detected with general additive mixed models, such as the summer-associated genus *Ferruginibacter.* The bottom row shows how relative abundance of the prevalence examples changes over time. While the same patterns are generally seen, they are more apparent for some taxa, like *Psychrobacter* and *Ferruginibacter*, and obscured for taxa like *Beijerinckiaceae* and Acinetobacter. Lines show local regression (locally estimated scatterplot smoothing [LOESS]).

Time was associated with decreased diversity and changes in community composition. Similarly, generally decreasing diversity with seasonal peaks in warmer summer months was observed over the series in the most abundant phyla: *Proteobacteria*, *Firmicutes*, *Actinobacteria*, *Bacteroidetes*, and *Cyanobacteria* (Fig. 5). Decreasing prevalence (the proportion of samples a group appears in) of the most abundant genera like Acinetobacter and *Bacillus* could help explain this trend, especially when coupled with taxa that became significantly less common, like *Beijerinckiaceae* (log odds = 0.24, *P* = 0.0004) (Fig. 5; see table S8 at doi.org/10.6084/m9.figshare.14211038). Many taxa were more frequently observed in the summer, such as *Ferruginibacter* (Fig. 5; see table S9 at doi.org/10.6084/m9.figshare.14211038), whose seasonal change in relative abundance was detected by fitting a 1-year cyclical spline as part of a general additive mixed model (*R*^2^ = 0.8, *P* = 0.00014), used previously to detect long-term and seasonal trends (51).

While community-level analyses capture the largest changes, they also obscure smaller changes. We were particularly interested in bacteria capable of surviving the harsh conditions of concrete, which would increase in relative abundance and/or prevalence throughout the series as other species die off. *Psychrobacter* was identified as an example of a taxon that increased throughout the series in both prevalence (log odds = 1.83, *P* = 0.017) and relative abundance (Fig. 5; see table S10 at doi.org/10.6084/m9.figshare.14211038).

### Earth Microbiome Project comparison.

The Earth Microbiome Project (EMP) is a data set of microbial communities from many different environments analyzed using a standardized protocol and classified in a standardized ontology (52). As little is known about the concrete microbiome, the EMP provides a simple comparison to similar environments and to potential influences such as aerosolic deposition or animal feces. Decontaminated concrete ASVs were trimmed to 90 bp and merged (vsearch at 99% similarity) with EMP ASVs to create a combined table of operational taxonomic units (OTUs).

**FIG 6:**
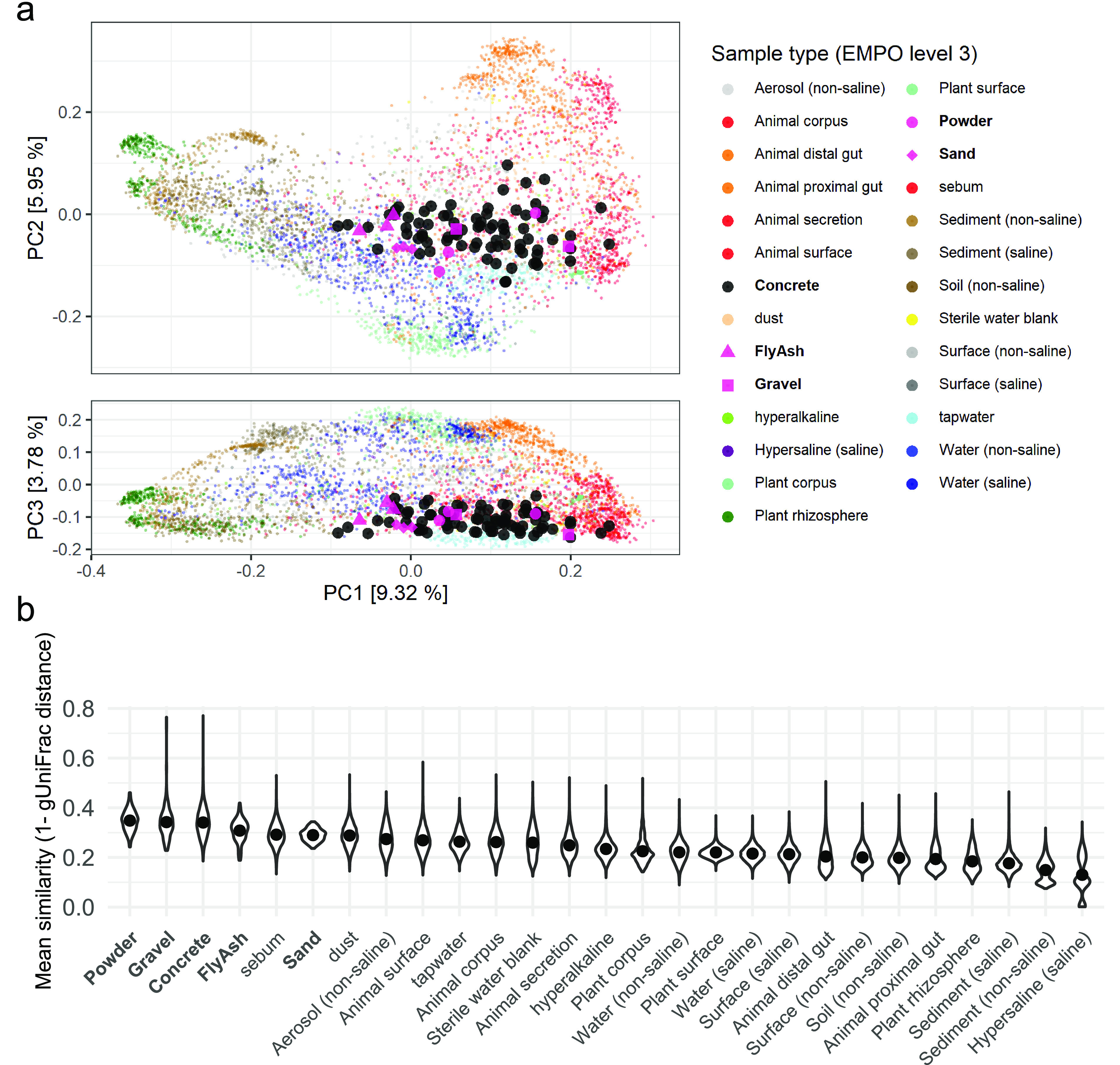
Concrete compared to Earth Microbiome Project samples. A merged OTU table was made with concrete ASVs clustered at 99% similarity with EMP ASVs (52) and tap water ASVs (48). (a) PCoA plot based on generalized (0.5) UniFrac distances. Concrete and precursor samples (bold) cluster together in the center of the PCoA plot, an area shared with a large diversity of other sample types that likely have many influences, such as animal surfaces, aerosols, surfaces, tap water, and alkaline environments. However, there is a clear spread from greater similarity to animal-related EMP samples (higher PC1 values) to environments like water and soils (lower PC1 values). (b) Similarity of concrete samples to precursor materials and EMPO3 groups shown with violin plots, where points represent the mean similarity.

Principal-coordinate analysis (PCoA) ordination of generalized (0.5) UniFrac distances shows that concrete communities overlap communities from aerosols, surfaces, negative controls (EMP sterile water blanks), sebum, dust, animal and corpus/surfaces, nonsaline waters, and hyperalkaline environments (Fig. 6a). Concrete samples are near the middle of PC1, between the major groups driving variance, with animal-associated communities found at high PC1 values and plant/soil communities at low PC1 values. However, concrete samples clearly have some spread along PC1, with most precursor samples found at lower, less animal-associated PC1 values. Concrete samples are also found at central PC2 values, above water samples. Concrete communities are more similar to nonsaline water and tap water communities than to saline water and plant corpus communities, which are separated along the PC3 axis.

Pairwise generalized (0.5) UniFrac similarities (similarity = 1 − distance) (53, 54) were used to assess the mean similarity of microbial communities for all concrete and EMP samples grouped at level 3 of the EMP ontology (EMPO) (55) (Fig. 6b). The communities observed in the cement powder and gravel used in the concrete mix were on average as similar to concrete communities as those of other concrete samples (∼0.34), with fly ash communities being slightly less similar (∼0.31). The most similar EMPO3 groups to concrete were sebum and dust, which were approximately as similar as the sand used in concrete production (∼0.29). Aerosols, animal surfaces, tap water, and sterile water blanks were the next most similar (∼0.26). Microbial communities from alkaline environments, plant and saline surfaces, and nonsaline water had a similarity of about 0.22, while sediment, soil, animal gut, and hypersaline environment microbial communities were the least similar to concrete communities. Changes in the similarity of concrete and EMP groups were also observed over time (Fig. S6).

10.1128/mSystems.01153-20.9 FIG S6 Concrete similarity to EMP groups over time. Concrete sample similarity (1 − generalized [0.5] UniFrac distances) to EMP groups is shown on the *y* axis, with months on the *x* axis. Points represent the mean similarity of a concrete sample to EMP samples in the indicated group. A linear regression is also plotted to highlight trends. Note that *y* axis scales vary between graphs. Samples are shaded by the average temperature of the 30 days before sampling to help indicate seasons. Download FIG S6, PDF file, 0.3 MB. Copyright © 2021 Kiledal et al. 2021 Kiledal et al. https://creativecommons.org/licenses/by/4.0/ This content is distributed under the terms of the Creative Commons Attribution 4.0 International license.

To characterize the effects of decontamination, similarities of concrete before decontamination to the EMP were also computed. Removing contaminant taxa increased the similarity of concrete sample communities to EMP samples from aerosol, plant rhizosphere, sebum, surface (saline, nonsaline, and plant), nonsaline water, dust, and nonsaline sediment communities (Fig. S7). It had little effect on similarity to saline water and sediment, hypersaline environments, hyperalkaline environments, animal guts and secretions, and tap water (Fig. S7). Decontamination also resulted in decreased similarity between concrete samples and all precursor materials except sand, which increased in similarity but remained the least similar precursor. Contaminant removal also decreased similarity to animal and plant corpuses, sterile water blanks, and animal surfaces, suggesting that they might be sources of contaminants.

10.1128/mSystems.01153-20.10 FIG S7 EMP similarity change due to decontamination. The effect of decontamination on concrete sample similarity (decontaminated similarity − contaminated similarity) to each EMP group, with points representing the mean similarity change and positive values indicating increased similarity following decontamination. Download FIG S7, PDF file, 0.2 MB. Copyright © 2021 Kiledal et al. 2021 Kiledal et al. https://creativecommons.org/licenses/by/4.0/ This content is distributed under the terms of the Creative Commons Attribution 4.0 International license.

Bacteria found in concrete were also found in many hyperalkaline and hypersaline EMP samples. The genus *Paenibacillus* was an indicator for hyperalkaline, plant rhizosphere, and aerosol EMP samples (IndVal = 0.63, *P* = 0.001), as was the genus *Bacillus* (IndVal = 0.60, *P* = 0.001) (see table S11 at doi.org/10.6084/m9.figshare.14211038). The genus *Halomonas* was an indicator for hypersaline, saline surface, and concrete samples (IndVal = 0.44, *P* = 0.001) (see table S11 at doi.org/10.6084/m9.figshare.14211038). At the ASV level, an *Exiguobacterium* sp. was an indicator of concrete and hyperalkaline samples (IndVal = 0.36, *P* = 0.001) while a *Halobacillus* indicated concrete and hypersaline samples (IndVal = 0.21, *P* = 0.009) and a member of *Rhodobacteraceae* found in concrete was an indicator for alkaline EMP samples (IndVal = 0.58, *P* = 0.001) (see table S4 at doi.org/10.6084/m9.figshare.14211038). We were also interested in bacteria uniquely associated with concrete and identified several indicators belonging to several genera: *Aliidiomarina* (IndVal = 0.44, *P* = 0.001), *Alishewanella* (IndVal = 0.34, *P* = 0.001), and *Chroococcidiopsis SAG 2023* (IndVal = 0.26, *P* = 0.005) (see table S11 at doi.org/10.6084/m9.figshare.14211038).

### Microbial bioindicators of ASR.

**FIG 7:**
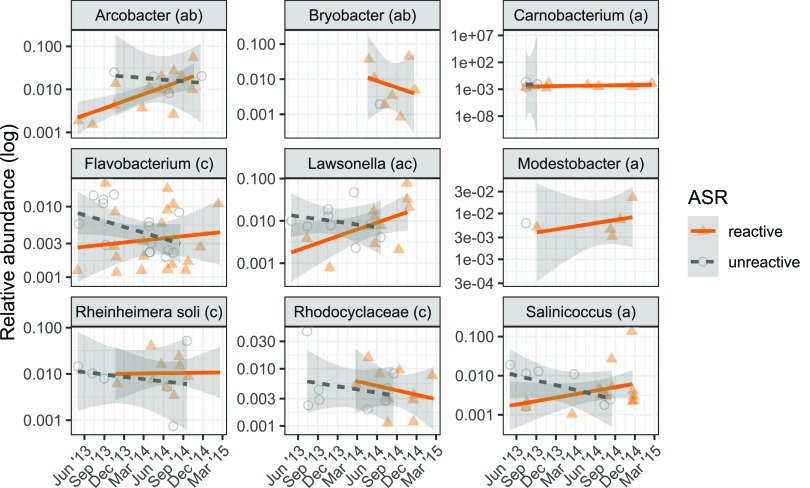
Potential bioindicators of the ASR. Bioindicators of the ASR determined with ssANOVA (indicated with the letter “a”) in *metagenomeSeq* (which determined time periods of differential abundance), indicator species analysis (indicated with the letter “b”) in *indicspecies* (which considers both specificity and sensitivity of the indicator), and logistic regression (indicated with the letter “c”) for presence/absence. All taxa are summarized at the genus level except for Rheinheimera soli and the family *Rhodocyclaceae*. For ssANOVA, indicator taxa that remained differentially abundant through the end of the series were identified. The following indicators were identified with ssANOVA: *Bryobacter* (interval, 10 to 21 months; *P* value, 0.004), *Sediminibacterium* (interval, 9 to 21 months; *P* value, 0.009), *Lawsonella* (interval, 16 to 21 months; *P* value, 0.016), *Arcobacter* (interval, 16 to 21 months; *P* value, 0.024), *Modestobacter* (interval, 12 to 21 months; *P* value, 0.027), *Salinicoccus* (interval, 19 to 21 months; *P* value, 0.031), and *Carnobacterium* (interval, 15 to 21 months; *P* value, 0.036). The following indicators were identified with indicator species analysis: *Arcobacter* (IndVal = 0.54, *P* = 0.007) and *Bryobacter* (IndVal = 0.39, *P*= 0.023). Logistic regression, run at multiple taxonomic levels, identified the following indicators: *Rhodocyclaceae* (log odds = 5.19, *P* = 0.016), Rheinheimera soli (log odds = 5.5, *P* = 0.020), *Flavobacterium* (log odds = 4.62, *P* = 0.046), and *Lawsonella* (log odds = 5.94, *P* = 0.038).

Taxa associated with ASR (Fig. 7) could potentially serve as bioindicators of the ASR. Several methods were used to identify bioindicator taxa grouped at the genus level, including indicator species analysis (46), smoothing spline analysis of variance (ssANOVA) from the *metagenomeSeq* R package (56), and logistic regression using presence/absence data. Indicator species analysis considers both the specificity and sensitivity of potential indicators, and it identified *Arcobacter* (IndVal = 0.54, *P* = 0.007) and *Bryobacter* (IndVal = 0.39, *P* = 0.023) as potential bioindicators of the ASR (Fig. 7).

In contrast, ssANOVA allows time to be explicitly considered in selecting indicators, as it finds time periods of differential abundance. As the ASR increases over time, we expected indicators to become or remain differentially abundant later in the series. ssANOVA identified the following potential indicators and their periods of differential abundance, with month 21 being the last in the series: *Bryobacter* (interval, 10 to 21 months; *P* = 0.004), *Sediminibacterium* (interval, 9 to 21 months; *P* = 0.009 [data not shown]), *Lawsonella* (interval, 16 to 21 months; *P* = 0.016), *Arcobacter* (interval, 16 to 21 months; *P* = 0.024), *Modestobacter* (interval, 12 to 21 months; *P* = 0.027), *Salinicoccus* (interval, 19 to 21 months; *P* = 0.031), and *Carnobacterium* (interval, 15 to 21 months; *P* = 0.036) (Fig. 7).

Finally, logistic regression was used on presence/absence data to find potential indicators with a significant (*P* < 0.05) effect for the interaction of time and ASR reactivity. Potential indicators of the ASR identified with logistic regression included *Rhodocyclaceae* (log odds = 5.19, *P* = 0.016), Rheinheimera soli (log odds = 5.5, *P = *0.020), *Flavobacterium* (log odds = 4.62, *P = *0.046), and *Lawsonella* (log odds = 5.94, *P = *0.038). In total, *Arcobacter*, *Lawsonella*, and *Bryobacter* were identified with multiple methods as robust potential bioindicators. The potential bioindicator taxa have increased abundance in ASR-reactive samples but, more importantly for use as indicators, are also found in more ASR-reactive samples than mitigated samples. Further, the differentiating power generally increases with time, as do the effects of the ASR.

## DISCUSSION

As the most-used building material in the world, concrete is a very common environment. However, relatively few microbes can survive this dry, salty, alkaline habitat with very low macronutrient concentrations. We predicted that the microbial community of concrete would be seeded from the component materials and that over time it would begin to resemble communities from other dry, saline, and/or alkaline environments. We further hypothesized that the physical and chemical changes due to ASR damage would be reflected in the microbial communities of ASR-reactive and ASR-mitigated concrete.

Here, we show that microbial communities in ASR-prone and ASR-mitigated concrete series reflected those of the components of concrete. The inclusion of fly ash to prevent ASR in the ASR-mitigated concrete mix, and the resulting difference in probability of ASR, did not have a statistically significant effect on overall community composition. However, individual taxa were identified with significantly different abundance patterns in the ASR-reactive and ASR-mitigated series, and these could potentially serve as microbial bioindicators of the ASR. As the cylinders in both series weathered, the diversity in their bacterial communities decreased, though we observed seasonal increases in diversity in the summer.

ASR generates a hygroscopic silica-rich gel, which, as it expands, cracks the concrete from the inside. We expected the ASR-reactive samples to be more alkaline than the ASR-mitigated samples, because fly ash reduces the pH (21). We also expected that because of the cracking, more water and nutrients would infiltrate the ASR-reactive samples. ASR reactivity, therefore, might increase selection for alkaliphiles while reducing selection for oligotrophs or xerophiles. In fact, the identification of *Bryobacter* as a potential bioindicator for the ASR was surprising, given its description as “mildly acidophilic” (57) and its previous association with wet environments: bogs (58), wetlands (59), and wastewater (60). Its presence may imply greater water infiltration into ASR-damaged concrete, though this genus has also been observed in desert biocrusts (61, 62). Another putative ASR bioindicator, *Modestobacter* (family *Geodermatophilaceae*), is commonly associated with dry environments: stone surfaces, desert biocrusts, and endolithic biofilms (63, 64). *Salinicoccus* is a member of the *Staphylococcaceae* commonly found in high-salt environments, including desert soils (65, 66), saline soils (67), coastal water (68), salt mines (69, 70), salterns (71), and soda lakes (72–74). These potential bioindicator genera are not known to be alkaliphiles. Alternatively, because concrete is seeded from several different materials, we also expected to see generalist species capable of surviving a variety of different stresses, as previously observed in a cultivation campaign (2). Several of the other potential ASR indicators identified, including *Arcobacter*, *Flavobacterium*, *Rheinheimera*, and *Rhodocyclaceae*, are generalists found in a wide range of environments. Interestingly, *Rheinheimera* spp. are found in marine (75), coastal (76–78), freshwater (79–81), industrial waste (82, 83), and alkaline environments (84, 85). One strain produces calcite (86)—potentially applicable for concrete biorepair—and another has recently been associated with stainless steel corrosion (87). The *Flavobacterium* and *Rhodocyclaceae* groups are true generalists (88, 89). Since the ASR-mitigated and ASR-reactive cylinders were produced using the same starting materials, with the exception of the fly ash, these bioindicators suggest that differences due to physical and chemical effects of the ASR are reflected in their microbial communities. Further work using genomics and metagenomics to analyze microbial metabolic functions in different types of concrete may better elucidate the physiological reasons for the differences.

Concrete samples share similarities with microbial communities in the Earth Microbiome Project (EMP) (52) from physically and chemically similar environments such as alkaline soils and travertine, desert soils, stone surfaces, and hypersaline lakes. Indicator species analysis with the combined EMP data found several bacterial groups strongly associated with both concrete and saline EMP samples, including the gammaproteobacterial genus *Halomonas* and the *Firmicutes* genus *Halobacillus*, which include both halotolerant and alkalitolerant species (90–92). Other groups of bacteria in the concrete community were strongly associated with alkaline environments, including *Paenibacillus*, *Bacillus*, *Exiguobacterium*, and *Rhodobacteraceae*. Several taxa were indicators of concrete, including halotolerant and alkalitolerant *Aliidiomarina* (93–95), *Alishewanella* (96, 97), and the desiccation-resistant cyanobacterium *Chroococcidiopsis* (98–100). We also observed several taxa in concrete that are commonly found in desert soil crusts (101, 102), including the radiation-resistant genera *Rubrobacter* (103) and Acinetobacter (104), the nitrogen-fixing genus *Frankia* (105), and the halophile- and alkaliphile-containing genera *Halomonas* (91, 106), *Bacillus* (107), and *Stenotrophomonas* (108, 109). Photosynthetic *Chloroflexi* (110) and *Cyanobacteria* belonging to the genus *Nostoc* (111, 112) are also common in saline soil crusts and were present in concrete. Common epilithic bacteria were also found in concrete, including *Sphingomonas*, *Frankiales*, *Truepera*, *Hymenobacter*, *Sphingobacteriales*, *Massilia*, *Stenotrophomonas*, and *Paenibacillus* (113–115). In addition, several species belonging to groups common in alkaline soda lakes were detected, including *Dietzia*, *Halomonas*, *Paenibacillus*, *Exiguobacterium*, *Bacillus*, and *Rhodobacteraceae* (116, 117). Fundamentally, these groups all tolerate osmotic stress, which is likely necessary for survival in concrete, whether it is imposed by salt concentration, pH, or low water activity.

Although each batch of concrete is seeded from its materials, the chemical and physical characteristics of all concrete are similar, which may impose strong enough selective pressure that after a time, microbial communities in concrete become broadly similar. Thus, we expected concrete to have a community of generally resilient microbes, with a few (poly)extremophiles, rather than an endemic community of unique microbes that have adapted to harsh conditions over a long period of time. Characteristics of the gravel (large aggregate) typically determine the ASR reactivity of the concrete (24), and 30 to 40% of the microbial community in these cylinders likely came from gravel. The contribution from cement powder (∼14% and 30% at the beginning and end of the study, respectively) was surprising, since it is a highly alkaline, oxidized material produced in high-temperature (∼1,500°C) furnaces (118). The gravel- and cement powder-associated microbes make up a larger portion of the microbial community in aged samples, suggesting that gravel and cement powder resemble concrete in terms of the constraints imposed on the microbial community. As the microbial community became less diverse, the survivors from these materials increased in relative abundance.

Unlike naturally occurring extreme environments, which are stable on time scales up to hundreds of thousands of years and allow ample time for evolution of specialists, concrete is unlikely to have endemic microbes. Instead, its microbial community is seeded from precursor materials in each batch and is also likely influenced and/or seeded by the surrounding environment, as suggested by summertime increases in diversity and DNA yield, a proxy for biomass (119). Seasonal changes in diversity and biomass are common in environments such as temperate soils and aerosols (120–122) and tend to be greater in stressed environments (123). In our samples, microbial growth or survival might have been enhanced in the summer due to greater precipitation, since infiltration of water would relieve xeric stress and could also bring dissolved nutrients into the concrete matrix. Further studies of older concrete, concrete exposed to different environmental conditions, or concrete subject to different types of damage will therefore have implications not just for bioindicators in the built environment but also for understanding microbial seeding of and adaptation to both concrete and other composite materials.

### Conclusions.

This work provides a comprehensive picture of microbial communities in concrete during the first 2 years of weathering. We discovered that most of the microbiome in concrete is seeded from the materials used to make it and that this microbial community is broadly similar to other communities exposed to different types of osmotic stress. We show that the community composition changes over the 2 years, decreasing in diversity over time, and that weather also impacted community composition, with increased diversity in summer months. Knowing how bacterial communities in and on concrete change over time potentially benefits several applications. Understanding the naturally resident bacteria and their temporal dynamics in concrete could improve ongoing efforts to repair concrete via microbially induced mineral deposition, and identification of indicator species of the concrete degrading alkali-silica reaction could enable earlier detection of the ASR and thus more effective damage mitigation.

## MATERIALS AND METHODS

### Preparation and exposure of test cylinders.

Two series of concrete cylinders (Fig. 1), one prone to ASR and the other having the risk of ASR mitigated with fly ash, were prepared in the UD Structures Lab using materials obtained from the Delaware Department of Transportation (DelDOT). ASR-reactive concrete was prepared following the mix design for DelDOT class A-503 concrete and contained 20.14 kg of cement, 6.76 kg of tap water, 26.94 kg fine aggregate (sand), and 47.40 kg large aggregate for a total weight of 101.24 kg. ASR-mitigated concrete was prepared using the mix design for DelDOT class A-3-50 with the same materials but with replacement of half of the cement with fly ash. ASR-mitigated concrete contained 15.38 kg each of cement and fly ash, 12.25 kg tap water, 46.31 kg fine aggregates, and 81.37 kg large aggregates for a total weight of 170.69 kg. Additional water was added to improve workability.

Concrete cylinders were produced using cylindrical plastic forms, 4 in. in diameter by 8 in. high, agitated with a shaker table after pouring to remove air pockets, and then capped and allowed to cure at room temperature for 2 weeks before removal from forms. One cylinder from each series was archived on 2 May 2013, and the rest were placed on the roof of Colburn Laboratory at the University of Delaware for exposure to weather while protected from foot and car traffic. One cylinder from each series was then archived every 4 to 8 weeks until 17 February 2015.

### Weather data.

Weather data for the sampling period were downloaded from Weather Underground for the KILG (New Castle Airport) site with the *rwunderground* R package. Summary weather information was compiled for the 30 days prior to each sampling. The R script used to generate summary information is included in the GitHub repository (github.com/MarescaLab/concrete_series).

### Sample processing, DNA extraction, 16S amplification, and sequencing.

From each cylinder, slices perpendicular to the long axis were obtained using a saw (blade and platform cleaned with 70% ethanol); a chisel cleaned with 70% ethanol was then used to remove the outside edges and break apart the section. Subsamples were pulverized using a ring and puck mill cleaned with 70% ethanol, and the resulting powder was stored at −20°C until DNA extraction. DNA was extracted in triplicate as described by Maresca et al. (2): 5 g of sample was washed with 20 ml of 0.5 M EDTA to remove divalent cations, followed by suspension in a lysis buffer and lysozyme with agitation at 37°C for 30 min. Proteinase K and 20% SDS were then added and incubated for 2.5 to 3 h with gentle agitation at 56°C, followed by chloroform (20 ml) extraction. One milliliter of 1.95 M sodium acetate was added to the aqueous phase of each sample and re-extracted with 0.8 volume of chloroform. Finally, DNA was precipitated with 1 volume of cold isopropanol and 0.1 volume 3 M sodium acetate, washed with 70% ethanol, allowed to dry, and resuspended in 200 μl Tris-EDTA (TE). DNA was reprecipitated with 13% polyethylene glycol (PEG) 8000, washed with 70% ethanol, dried, resuspended in 25 μl 10 mM Tris (pH 8.0), and stored at −20°C.

DNA was also extracted in triplicate from equivalent quantities (5 g) of precursor materials used in concrete cylinder production: large aggregates (gravel), fine aggregates (sand), Portland cement, and fly ash. Triplicate negative-control DNA extractions were also performed on glass beads (5 g) that had been sterilized by bleaching, UV irradiation, and autoclaving. Extracted DNA was quantified with an Invitrogen Qubit 2.0 fluorometer. PCR with general 16S primers 357F and 806R and visualization with gel electrophoresis were performed to check that no PCR inhibitors were present. 16S amplicon libraries were generated at the UD Sequencing and Genotyping Center following the Illumina 16S metagenomic sequencing library preparation protocol (124) using primers 357F (CCTACGGGNGGCWGCAG) and 806R (GACTACHVGGGTATCTAATCC) targeting the V3-V4 region of the 16S gene (29, 30). Libraries were sequenced with an Illumina MiSeq system using 2 × 300-bp paired-end reads. One outlier sample (021715U_6) was discarded at this point because only 1,313 reads were obtained, while all other samples had >50,000 reads.

### Determination of ASVs, taxonomy assignment, and phylogenetic placement.

Primers were trimmed from reads using Cutadapt (v. 1.18) (125) with the following parameters: -g, CCTACGGGNGGCWGCAG; -a, GGATTAGATACCCBDGTAGTC; -G, GACTACHVGGGTATCTAATCC; -A, CTGCWGCCNCCCGTAGG; -minimum-length, 50, -n, 2. Exact amplicon sequence variants were determined using the DADA2 denoiser (32) plugin for QIIME 2 (version 2019.10) (31) with the following parameters: p-trunc-len-f = 270, p-trunc-len-*r* = 191, and p-max-ee = 2. ASVs are more specific than commonly used operational taxonomic units (OTUs), which consist of sequences clustered by similarity (typically 97%). DADA2 infers exact ASVs, merges paired-end reads, removes chimeric sequences, and generates a per-sample count table of ASV observations.

Taxonomy was assigned to ASVs using the QIIME 2 naive Bayes machine-learning feature classifier (126) trained on the Greengenes (127) and SILVA (release 132) (34) databases trimmed to match the V3-V4 region sequenced. Trees were constructed using SEPP (35, 36) to place ASV representative sequences into the SILVA reference phylogeny (release 128) (34). This approach was developed to overcome difficulties in *de novo* tree construction from short sequencing fragments and facilitates comparison of amplicon data from different variable regions (36).

### Pairwise ASV comparisons.

Correlation, or analogous metrics in the case of SPIEC-EASI and *propr*, were computed using SPARCC (40) implemented in FastSpar (41) with the parameters iterations = 50 and permutations = 1,000, the SPIEC-EASI R package (42) with the parameters method = ‘glasso’, lambda.min.ratio = 1e−3, nlambda = 30, and rep.num = 50, and the Propr R package (43) with the parameters metric = “rho.” and *P* = 999. SPARCC *P* values were computed by permutation, and only correlations with an absolute value greater than 0.35 (the default cutoff) and *P* values of <0.05 were considered. In addition to the inherent error control in SPIEC-EASI, pseudo-*P* values were computed as 1-edge stability across the sparsity path. Only edges with a pseudo-*P* of ≤0.5 were used. Only *propr* rho values of >0.65 and <−0.5 were considered, in line with recommended false discovery rate (FDR) control.

Levenshtein edit distances for sequence similarity were computed for each ASV pair using the *stringdistmatrix* function of the *stringdist* R package (128).

Chi-square tests were used to compare distributions for each ASV pair using the *chisq.test* function in the R stats package (129). *P* values were simulated with 100,000 permutations.

### Contaminant identification.

Contaminant ASVs were identified with the *prevalence* method of the *decontam* package (39) with a threshold of 0.33, corresponding to a probability threshold below which the null hypothesis (noncontaminant) is rejected in favor of the alternative hypothesis (contaminant).

Sequences with a BLAST similarity of >99% to unique lab strains were identified as contaminants. Correlation analyses were used to identify additional suspected lab contaminants.

Using pairwise ASV correlations calculated as described above, ASVs highly correlated with identified contaminants were also deemed to be contaminants. Two groups of contaminants were used as starting points: a cluster of highly intercorrelated ASVs present in negative controls (calculated independently with each correlation-like metric [sparcc # of pos - neg >0 and mean cor > 0.3; spiec easi # pos - neg > 0; propr # pos - neg > 0]) and lab contaminants identified by BLAST similarity as described above. For both contaminant starting groups, highly correlated ASVs were separately identified as contaminants with each of the correlation-like metrics (reagent: sparcc # pos > 10 & mean cor > 0.25; spiec-easi # pos - neg >5; propr # pos - neg > 0; lab: sparcc # pos > 1 & mean > 0.3; spiec-easi # pos - neg > 5, propr # pos > 0). For each metric, a secondary search for highly correlated ASVs was then conducted against all contaminants identified up to this point (sparcc mean > 0.3 & # pos > 5; spiec-easi # pos > 5; propr mean > 0.3 & # pos > 1). Cutoffs were chosen based on plotted distributions of net positive correlations versus mean correlation. ASVs found only in negative-control samples were also classified as contaminants.

To account for residual sequencing errors (baseline Illumina error rates, ∼0.0042 error per base; DADA2 residual error rate, ∼2.5 × 10^−8^), ASVs only one nucleotide edit away (Levenshtein edit distance, calculated as described above) (32, 130) from identified contaminants were also classified as contaminants and assigned the same detection method as their match.

### Statistical analysis.

Within sample (alpha) diversity metrics Faith’s PD and Shannon were calculated with the QIIME 2 Diversity Plugin (31). Changes in alpha diversity over time were evaluated with linear mixed-effect models [alpha.div ∼ scaled_months + scaled_avg_temperature + ASR_status + (1 | ASR_status)] using the *lmer* function of the *lme4* R package (131) and *lmerTest* package to obtain *P* values (132).

The between-sample (beta) diversity metrics Bray-Curtis, Jaccard, UniFrac, weighted UniFrac (53), and generalized UniFrac (54) were also calculated with the QIIME 2 diversity plugin (31). Permutational multivariate analysis of variance was performed with the *adonis2* function of the *vegan* R package (50).

Principal-coordinate analysis was performed using the QIIME 2 diversity plugin (31).

Indicator species analysis was used to determine indicators of ASR status, implemented in the *multipatt* function of the *indicspecies* R package (version 1.7.8) (46) using the IndVal.g association statistic and 1,000 permutations. Taxonomic agglomeration at the genus level was performed using the *tax_glom* function of the *speedyseq* (133) R package. Only genera with more than 5 observations in more than 5 samples were considered.

Leveraging this study’s longitudinal design, the *fitMultipleTimeSeries* function of the *MetagenomeSeq* R package (version 1.28.0) (134, 135) applied ssANOVA to identify ASVs with differential abundance that increased throughout the experiment (formula, abundance ∼ time × ASR status; permutations, 1,000). Only genera with more than 5 observations in more than 5 samples were considered.

The ASV observation table was converted to presence/absence, and logistic regression was performed with the *glm* function of the R *stats* package using family = “binomial” and the formula presence/absence ∼ ASR × scale (months). This model was applied to taxa summarized at all levels that were observed in at least 5 samples.

Following methods described in reference 51, general additive mixed models were applied with the *mgcv* R package (136). Abundances were Hellinger transformed prior to running the model *scaled.abundance* ∼ *s*(DoY, *bs* = “*cc*,” *k* = 4) + *s*(months, *bs* = “*cr*,” *k* = 1). The cyclic cubic regression spline for day of year detects seasonal changes in abundance, limited in complexity to 4 knots. In addition, time of year with peak abundance can be identified by inspection of this spline. The cubic regression spline limited to 1 knot is essentially a linear regression, which models changes occurring over the entire length of the series. This model was applied to taxa summarized at all levels that were observed in at least 5 samples.

To understand potential sources of bacteria in concrete samples, SourceTracker2 (47, 137) was used to calculate source mixing proportions for each concrete sample (alpha1 = 0.01, alpha2 = 1). Sources were pooled and subsampled to a depth of 8,000, while sink samples were subsampled to a depth of 635.

### Comparison to Earth Microbiome Project.

Comparison to the EMP is limited to broad comparisons (36) due to different primer biases of the EMP V4 region primers 515F and 806R and the V3V4 primers 357F and 806R used here (138, 139) and the short amplicon length (90 bp) from early EMP samples. The 90-bp release 1 of the Earth Microbiome Project was downloaded from ftp://ftp.microbio.me/emp/release1/otu_tables/deblur/emp_deblur_90bp.release1.biom. From this, an expanded version of the standard 5k sample EMP subset was produced, with additional inclusion of sterile water blanks and samples from highly alkaline environments. Representative sequences were extracted from the OTU table for import into QIIME 2. Additional samples from a study of tap water (48) prepared with the EMP protocol were included; representative sequences and a BIOM table generated from reads trimmed to 90 bp and processed with deblur (140) were downloaded from Qiita (141) study 10251.

Representative sequences for ASVs from the current study were trimmed to the same 90 bp as EMP and tap water samples using the GetV4Region.py script released by the EMP authors. After tables and representative sequences were merged, the QIIME 2 VSEARCH plugin (33) was used to cluster reads from this study, tap water samples, and EMP samples at 99% nucleotide similarity. The 99% similarity threshold was chosen to account for slight variability but was set higher than the standard 97% due to the short sequence length and because both EMP reads and those from this study were previously denoised.

Combined EMP, tap water, and concrete tables were produced for raw concrete samples, “decontaminated” concrete samples, and only contaminants of concrete samples, allowing evaluation of concrete decontamination. Sequences from this combined data set were inserted into release 128 of the SILVA reference phylogeny using SEPP (35). Taxonomy was assigned to ASVs using a naive Bayes classifier trained on the 515F-806R region of the SILVA 132 release.

QIIME 2 was used to calculate distance tables based on several diversity metrics (Bray-Curtis, Jaccard, UniFrac, weighted UniFrac, and generalized [0.5] UniFrac) and corresponding PCoA ordinations.

Indicator species analysis was conducted with the *multipatt* function of the *Indicspecies* R package (46). EMPO level 3 groups were used for groupings with 999 permutations and max.order = 3. This analysis was also conducted at the genus level with prior taxonomic agglomeration using the *tax_glom* function of the *speedyseq* (133) R package.

The relative occurrence frequency was calculated as described by Thompson et al. (52) using custom R scripts available at github.com/MarescaLab/concrete_series. Per-ASV Shannon entropies of the relative occurrence frequencies were calculated with the *diversity* function of the *vegan* R package (50).

### Figure plotting.

Figures 1, 4, and 7 and many of the supplemental figures were produced with the ggplot2 (142) R package. Heat trees (Fig. 2; Fig. S2 and S3) were produced using the Metacoder (143) R package.

### Data availability.

Data processing and analysis scripts are available at github.com/MarescaLab/concrete_series. Demultiplexed 16S amplicon data sets trimmed of Illumina adapters, but otherwise unmodified, were deposited at the NCBI Sequence Read Archive (SRA) under the BioProject number PRJNA629592. For tables S1 to S4 and S8 to S11 (tsv files), see doi.org/10.6084/m9.figshare.14211038.
