# Supplementary figures and images for: Bacterial Communities in Concrete Reflect Its Composite Nature and Change with Weathering
Source: mSystems. 2021 May 4;6(3):e01153-20. doi: 10.1128/mSystems.01153-20 (PMC8269252; doi:10.1128/mSystems.01153-20)

**A)**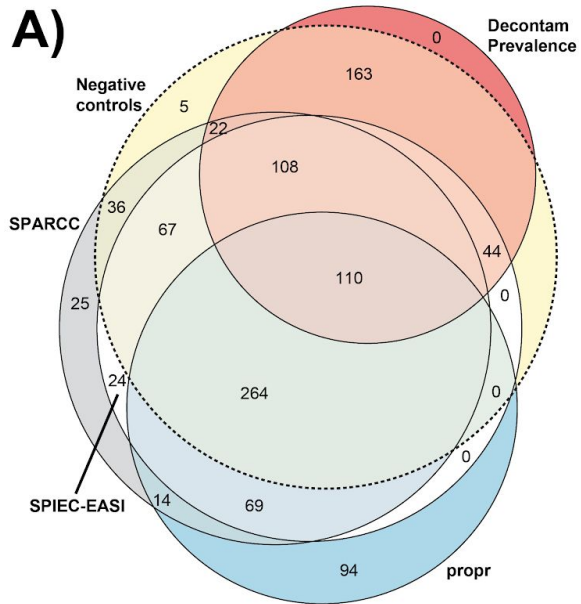**B)**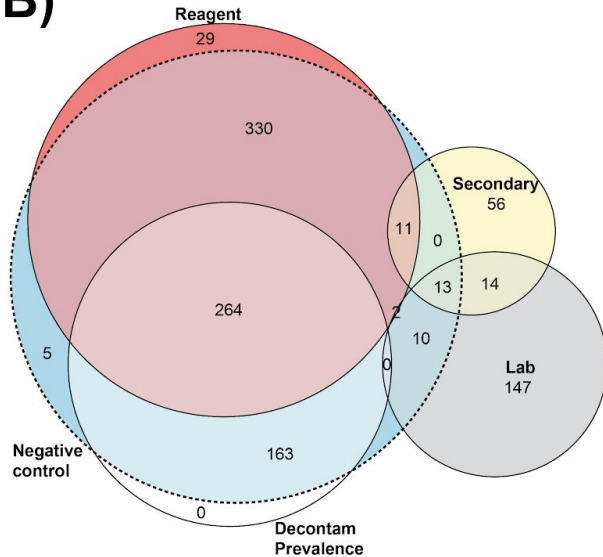

Supplement: FIG S1 [file msystems.01153-20-sf001.pdf]

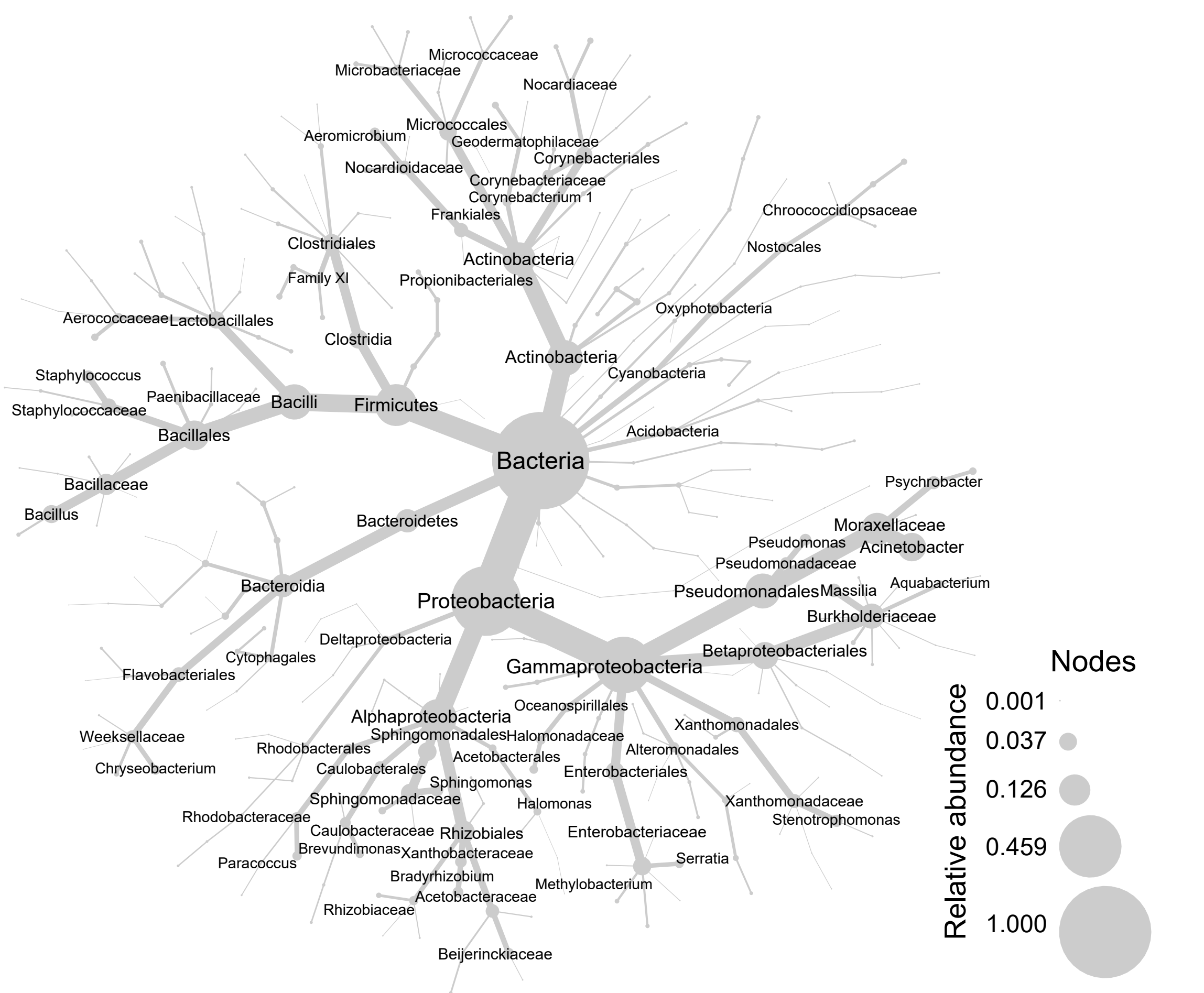

Supplement: FIG S2 [file msystems.01153-20-sf002.pdf]

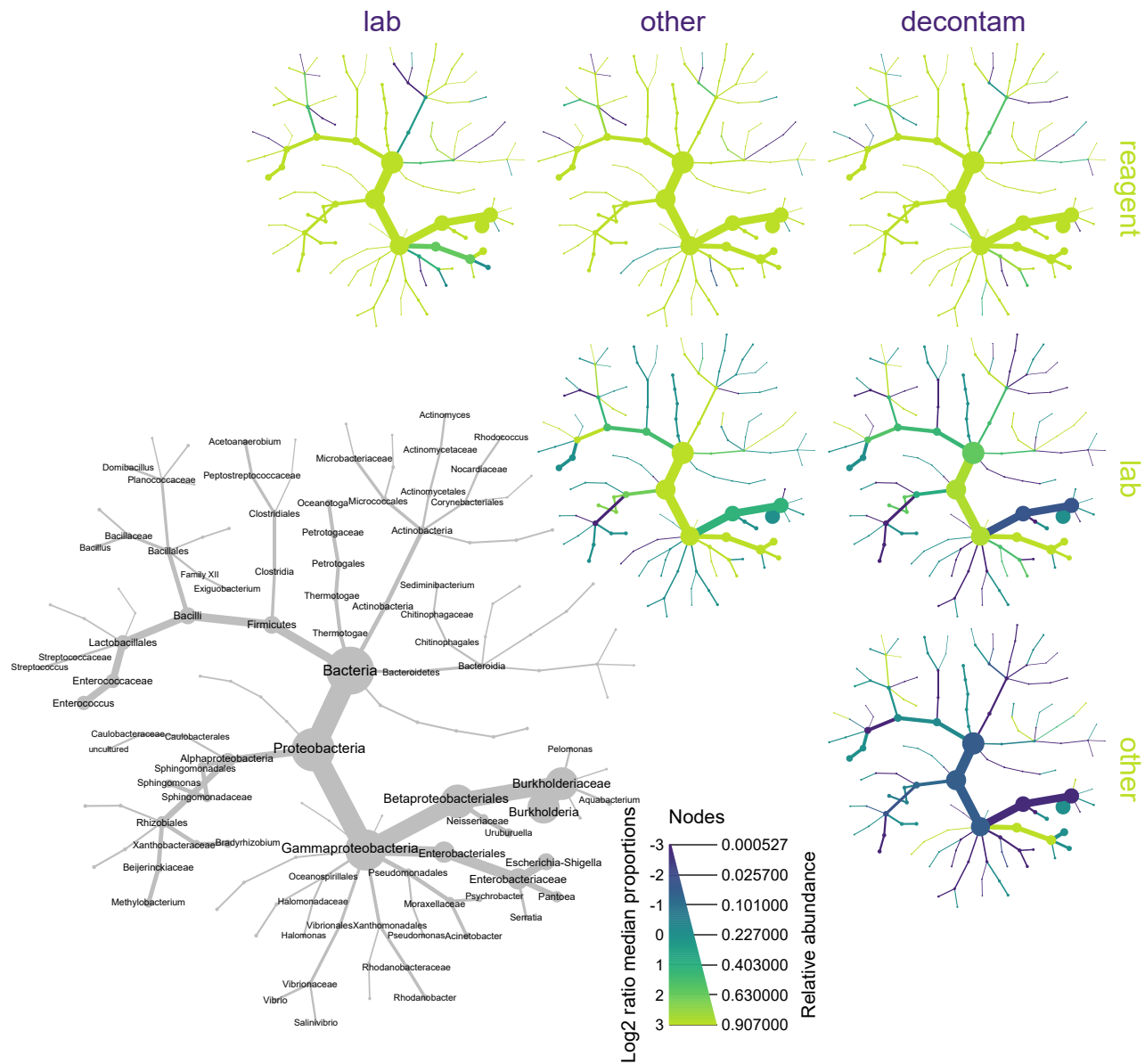

Supplement: FIG S3 [file msystems.01153-20-sf003.pdf]

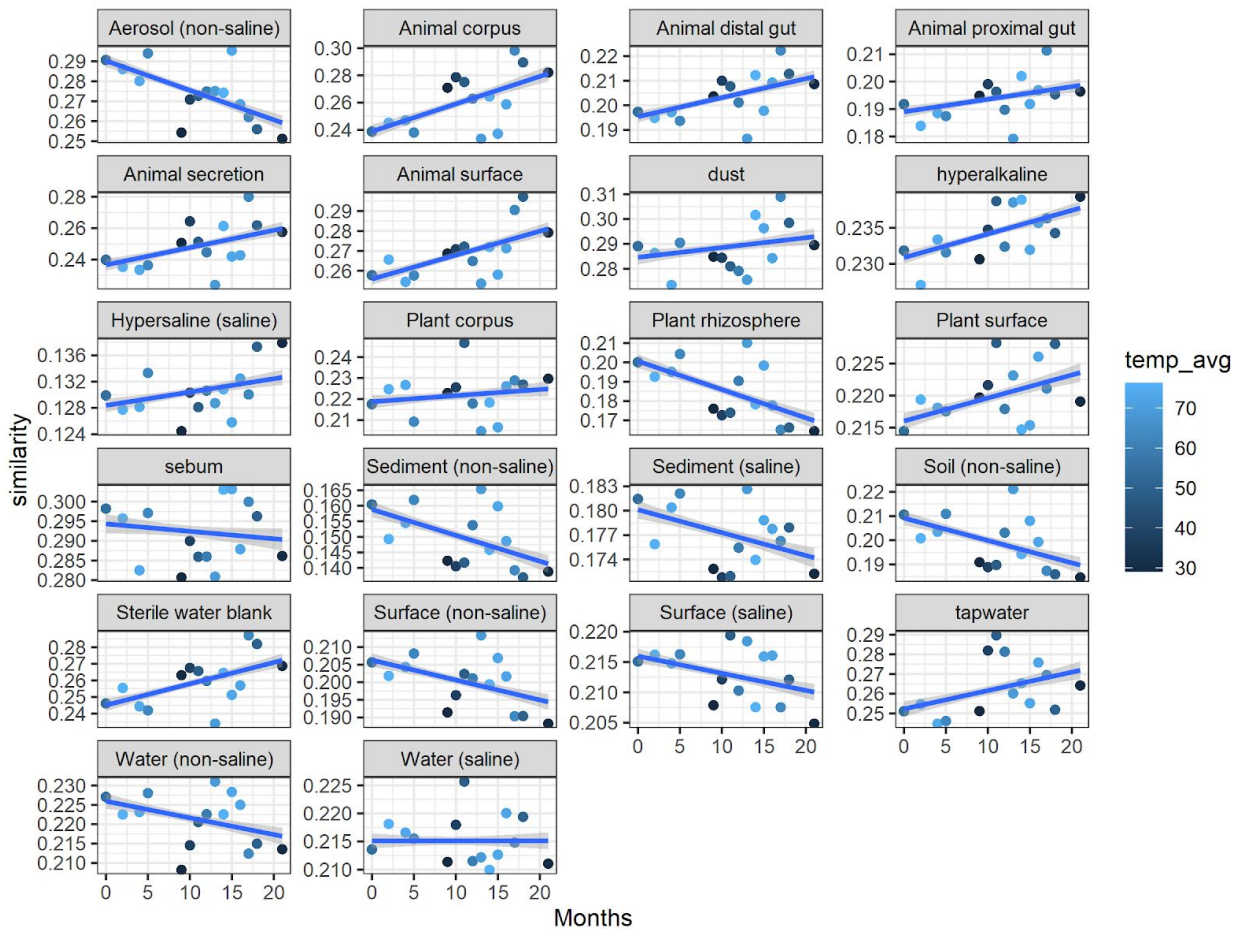

Supplement: FIG S6 [file msystems.01153-20-sf006.pdf]

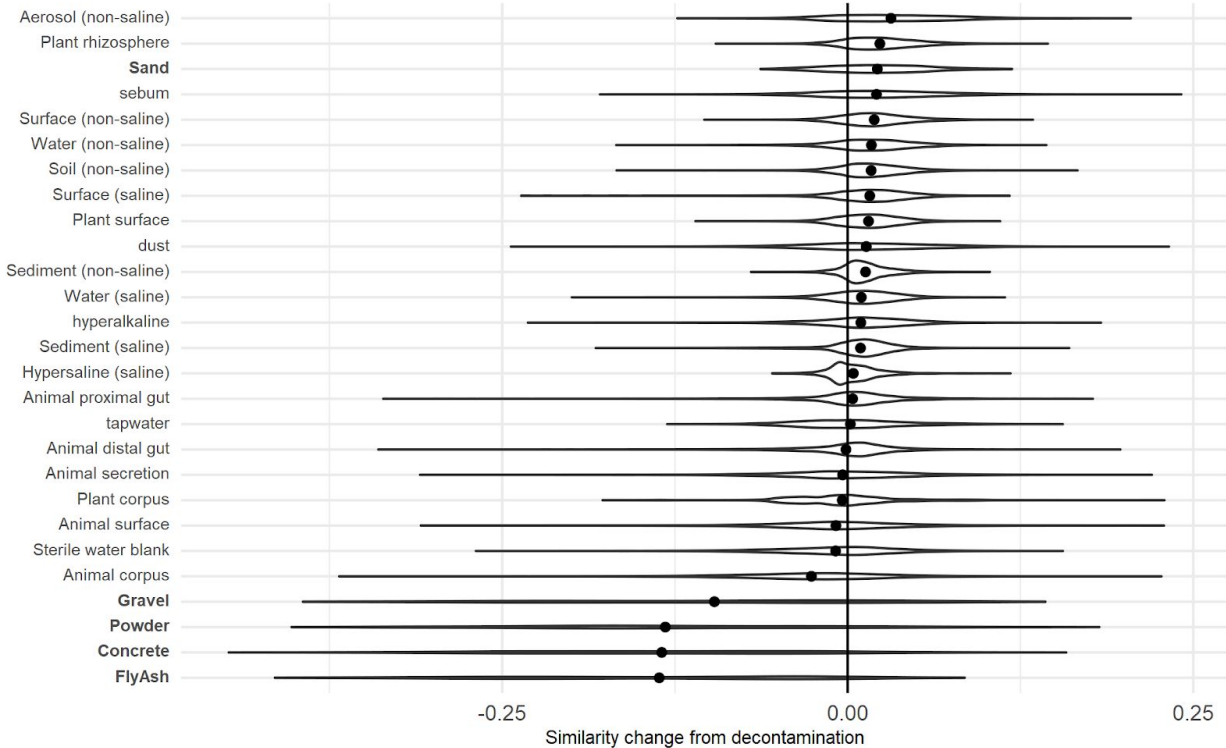

Supplement: FIG S7 [file msystems.01153-20-sf007.pdf]
