# Supplementary material for: Bacterial Communities in Concrete Reflect Its Composite Nature and Change with Weathering
Source: mSystems. 2021 May 4;6(3):e01153-20. doi: 10.1128/mSystems.01153-20 (PMC8269252; doi:10.1128/mSystems.01153-20)

a

Shannon diversity

Coefficient	Estimate	p.value
Months	0.0153	0.913
ASRreactive	0.1040	0.622
Temp	-0.0779	0.444
Months:ASRreactive	-0.1370	0.493

d

Faith's PD

Coefficient	Estimate	p.value
Months	-1.600	0.201
ASRreactive	1.570	0.456
Temp	1.760	0.0537
Months:ASRreactive	0.282	0.874

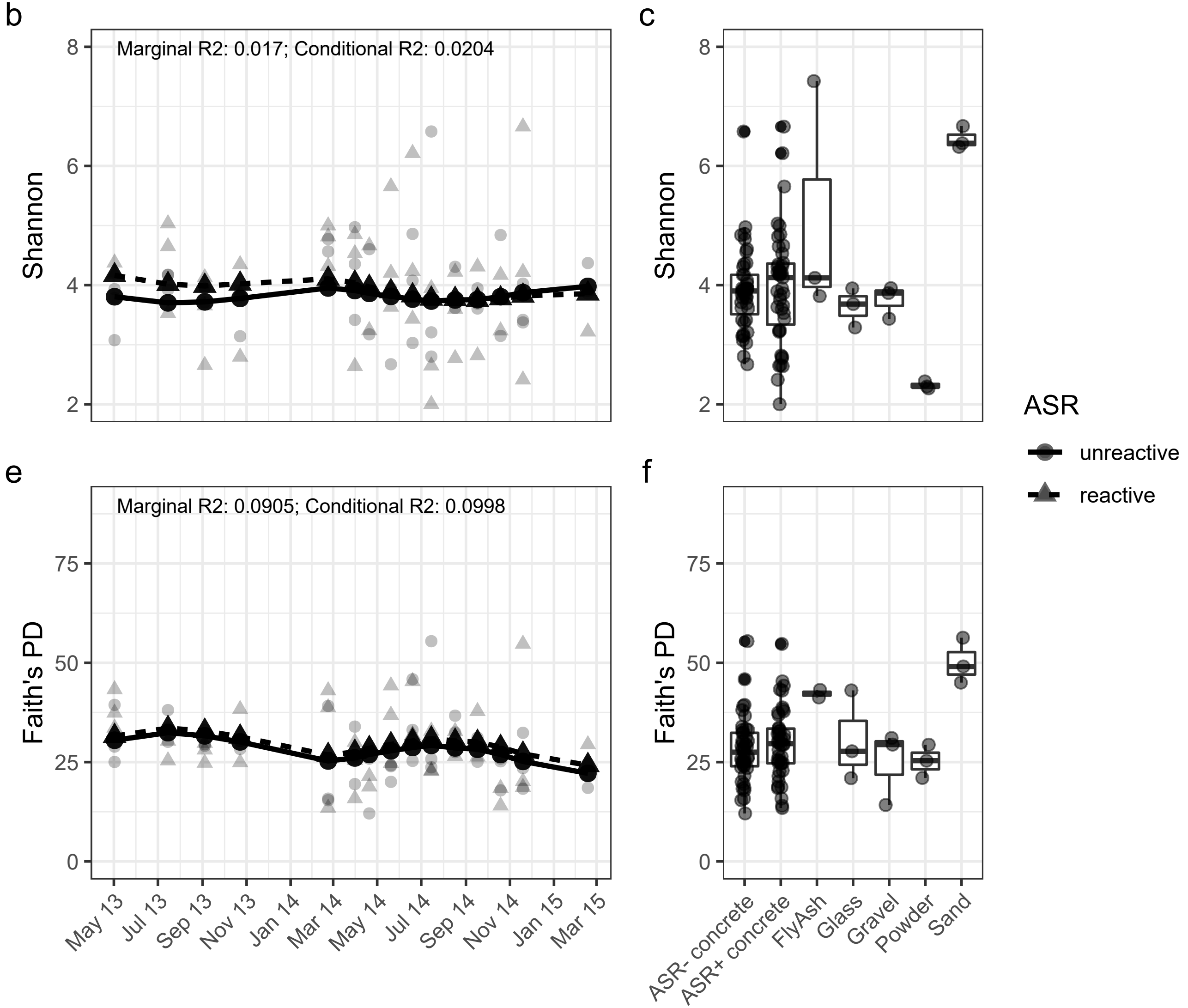

Supplement: FIG S4 [file msystems.01153-20-sf004.pdf]
