# Supplementary material for: Bacterial Communities in Concrete Reflect Its Composite Nature and Change with Weathering
Source: mSystems. 2021 May 4;6(3):e01153-20. doi: 10.1128/mSystems.01153-20 (PMC8269252; doi:10.1128/mSystems.01153-20)
Supplement: FIG S5 [file msystems.01153-20-sf005.pdf]

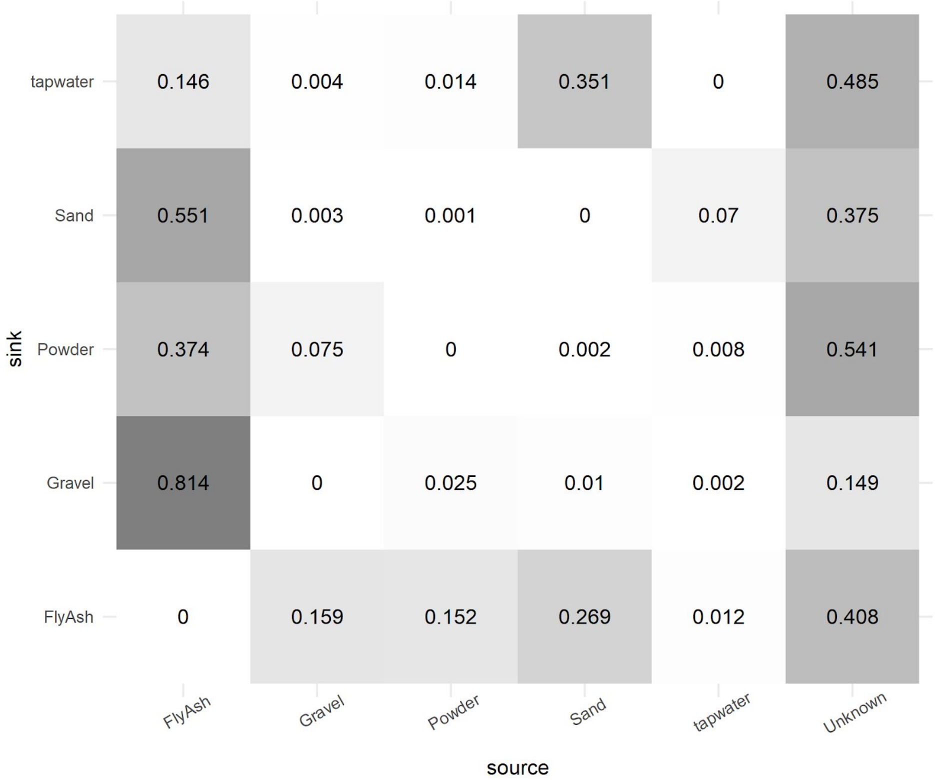
sink
tapwater
Sand
Powder
Gravel
FlyAsh
0.146
0.004
0.014
0.351
0
0.485
0.551
0.003
0.001
0
0.07
0.375
0.374
0.075
0
0.002
0.008
0.541
0.814
0
0.025
0.01
0.002
0.149
0
0.159
0.152
0.269
0.012
0.408
FlyAsh
Gravel
Powder
Sand
tapwater
Unknown
source
