# Supplementary material for: Bacterial Communities in Concrete Reflect Its Composite Nature and Change with Weathering
Source: mSystems. 2021 May 4;6(3):e01153-20. doi: 10.1128/mSystems.01153-20 (PMC8269252; doi:10.1128/mSystems.01153-20)
Supplement: TABLE S1 [file msystems.01153-20-st001.pdf]

Metric	Terms Tested	Coefficient	R2	F	p.value	Dispersion p.value
Bray-Curtis	Sequentially	Temperature	0.019	1.502	0.004**	0.487
Bray-Curtis	Sequentially	Months	0.02	1.539	0.002**	0.487
Bray-Curtis	Sequentially	ASR	0.01	0.754	0.991	0.307
Bray-Curtis	Marginally	Temperature	0.019	1.463	0.005**	0.487
Bray-Curtis	Marginally	Months	0.019	1.518	0.002**	0.487
Bray-Curtis	Marginally	ASR	0.01	0.754	0.996	0.307
