# Supplementary material for: Bacterial Communities in Concrete Reflect Its Composite Nature and Change with Weathering
Source: mSystems. 2021 May 4;6(3):e01153-20. doi: 10.1128/mSystems.01153-20 (PMC8269252; doi:10.1128/mSystems.01153-20)
Supplement: TABLE S2 [file msystems.01153-20-st002.pdf]

Dist. metric	Coefficient(s)	Pearson r	*P*-value
gUniFrac	Months	0.071	0.051
gUniFrac	Temperature	0.122	0.002**
gUniFrac	Months, Temperature	0.134	0.001***
BrayCurtis	Months	0.033	0.156
BrayCurtis	Temperature	0.09	0.003**
BrayCurtis	Months, Temperature	0.078	0.006**
