# Supplementary material for: Bacterial Communities in Concrete Reflect Its Composite Nature and Change with Weathering
Source: mSystems. 2021 May 4;6(3):e01153-20. doi: 10.1128/mSystems.01153-20 (PMC8269252; doi:10.1128/mSystems.01153-20)
Supplement: TABLE S3 [file msystems.01153-20-st003.pdf]

Metric	Terms Tested	Coefficient	R2	F	p.value	Dispersion p.value
gUniFrac	Sequentially	Temperature	0.022	2.005	0.029*	0.595
gUniFrac	Sequentially	Months	0.022	1.931	0.03*	0.595
gUniFrac	Sequentially	ASR	0.009	0.843	0.586	0.231
gUniFrac	Marginally	Temperature	0.02	1.838	0.033*	0.595
gUniFrac	Marginally	Months	0.021	1.928	0.036*	0.595
gUniFrac	Marginally	ASR	0.009	0.843	0.58	0.231
